# Perioperative Modified FOLFIRINOX for Resectable Pancreatic Cancer: A Nonrandomized Controlled Trial

**DOI:** 10.1001/jamaoncol.2024.1575

**Published:** 2024-06-20

**Authors:** Michael Cecchini, Ronald R. Salem, Marie Robert, Suzanne Czerniak, Ondrej Blaha, Daniel Zelterman, Moein Rajaei, Jeffrey P. Townsend, Guoping Cai, Sumedha Chowdhury, Deanne Yugawa, Robert Tseng, Carlos Mejia Arbelaez, Jingjing Jiao, Kenneth Shroyer, Jaykumar Thumar, Jeremy Kortmansky, Wajih Zaheer, Neal Fischbach, Justin Persico, Stacey Stein, Sajid A. Khan, Charles Cha, Kevin G. Billingsley, John W. Kunstman, Kimberly L. Johung, Christina Wiess, Mandar D. Muzumdar, Erik Spickard, Vasily N. Aushev, George Laliotis, Adham Jurdi, Minetta C. Liu, Luisa Escobar-Hoyos, Jill Lacy

**Affiliations:** 1 Department of Internal Medicine (Medical Oncology), Yale University School of Medicine, New Haven, Connecticut; 2 Department of Surgery, Yale University School of Medicine, New Haven, Connecticut; 3 Department of Pathology, Yale University School of Medicine, New Haven, Connecticut; 4 Department of Radiology, Yale University School of Medicine, New Haven, Connecticut; 5 Department of Biostatistics, Yale School of Public Health, New Haven, Connecticut; 6 Department of Therapeutic Radiology, Yale School of Public Health, New Haven, Connecticut; 7 Department of Pathology, Renaissance School of Medicine, Stony Brook University, Stony Brook, New York; 8 Yale Cancer Center, Yale University School of Medicine, New Haven, Connecticut; 9 Natera Inc, Austin, Texas

## Abstract

**Importance:**

Pancreatic ductal adenocarcinoma (PDAC) is an aggressive malignant tumor, and durable disease control is rare with the current standard of care, even for patients who undergo surgical resection.

**Objective:**

To assess whether neoadjuvant modified 5-fluorouracil, leucovorin, oxaliplatin, and irinotecan (mFOLFIRINOX) leads to early control of micrometastasis and improves survival.

**Design, Setting, and Participants:**

This open-label, single-arm, phase 2 nonrandomized controlled trial for resectable PDAC was conducted at the Yale Smilow Cancer Hospital from April 3, 2014, to August 16, 2021. Pancreatic protocol computed tomography was performed at diagnosis to assess surgical candidacy. Data were analyzed from January to July 2023.

**Interventions:**

Patients received 6 cycles of neoadjuvant mFOLFIRINOX before surgery and 6 cycles of adjuvant mFOLFIRINOX. Whole blood was collected and processed to stored plasma for analysis of circulating tumor DNA (ctDNA) levels. Tumors were evaluated for treatment response and keratin 17 (K17) expression.

**Main Outcomes and Measures:**

The primary end point was 12-month progression-free survival (PFS) rate. Additional end points included overall survival (OS), ctDNA level, tumor molecular features, and K17 tumor levels. Survival curves were summarized using Kaplan-Meier estimator.

**Results:**

Of 46 patients who received mFOLFIRINOX, 31 (67%) were male, and the median (range) age was 65 (46-80) years. A total of 37 (80%) completed 6 preoperative cycles and 33 (72%) underwent surgery. A total of 27 patients (59%) underwent resection per protocol (25 with R0 disease and 2 with R1 disease); metastatic or unresectable disease was identified in 6 patients during exploration. Ten patients underwent surgery off protocol. The 12-month PFS was 67% (90% CI, 56.9-100); the median PFS and OS were 16.6 months (95% CI, 13.3-40.6) and 37.2 months (95% CI, 17.5-not reached), respectively. Baseline ctDNA levels were detected in 16 of 22 patients (73%) and in 3 of 17 (18%) after 6 cycles of mFOLFIRINOX. Those with detectable ctDNA levels 4 weeks postresection had worse PFS (hazard ratio [HR], 34.0; 95% CI, 2.6-4758.6; *P* = .006) and OS (HR, 11.7; 95% CI, 1.5-129.9; *P* = .02) compared with those with undetectable levels. Patients with high K17 expression had nonsignificantly worse PFS (HR, 2.7; 95% CI, 0.7-10.9; *P* = .09) and OS (HR, 3.2; 95% CI, 0.8-13.6; *P* = .07).

**Conclusions and Relevance:**

This nonrandomized controlled trial met its primary end point, and perioperative mFOLFIRINOX warrants further evaluation in randomized clinical trials. Postoperative ctDNA positivity was strongly associated with recurrence. K17 and ctDNA are promising biomarkers that require additional validation in future prospective studies.

**Trial Registration:**

ClinicalTrials.gov Identifier: NCT02047474

## Key Points

**Question:**

Does perioperative 5-fluorouracil, leucovorin, oxaliplatin, and irinotecan (FOLFIRINOX) improve survival in patients with resectable pancreatic ductal adenocarcinoma (PDAC)?

**Findings:**

In this single-arm phase 2 nonrandomized controlled trial for resectable PDAC including 46 patients, the 12-month progression-free survival (PFS) was 67%, which met the primary end point of a 12-month PFS of 50% or greater. Detectable circulating tumor DNA levels, high tumor keratin 17 expression, and mutational signature SBS15 were associated with decreased survival.

**Meaning:**

In this study, perioperative modified FOLFIRINOX was safe and effective, with a clinically meaningful improvement in survival compared with historical controls.

## Teaser

This nonrandomized controlled trial assesses whether neoadjuvant modified 5-fluorouracil, leucovorin, oxaliplatin, and irinotecan (FOLFIRINOX) leads to early control of micrometastasis and improves survival.

## Introduction

Pancreatic ductal adenocarcinoma (PDAC) remains one of the most lethal malignant tumors and is predicted to be the second leading cause of cancer-related death in the US by 2030.^1^ There have been few major advances in treatment for patients with PDAC over the past decade, and it remains a cancer of significant unmet need. Most patients with PDAC present with locally advanced unresectable or metastatic disease and are not candidates for curative therapy. However, approximately 15% to 20% of patients are diagnosed with surgically resectable disease, which is potentially curable with surgery and adjuvant chemotherapy.^2,3^ Unfortunately, most patients with resectable PDAC will develop recurrent disease and ultimately die of advanced PDAC.

For metastatic PDAC, 5-fluorouracil, leucovorin, irinotecan, and oxaliplatin (FOLFIRINOX) is the most active chemotherapy with the longest survival as initial treatment.^4^ In 2018, 6 months of adjuvant FOLFIRINOX was demonstrated to be superior to adjuvant gemcitabine for resected PDAC and was established as the standard of care.^5^ However, long-term follow-up reveals that only 26.1% of patients remained disease free at 5 years after adjuvant FOLFIRINOX.^6^ There is strong rationale for incorporation of neoadjuvant FOLFIRINOX into the management of resectable PDAC. For example, active systemic therapy preoperatively presents an opportunity to eradicate occult micrometastatic disease early in the disease course, while pathologic downstaging may improve surgical outcomes and lower locoregional recurrence.^7,8^ Neoadjuvant therapy also provides a test of biologic behavior and spares patients with extremely aggressive disease a futile operation. Furthermore, given the complexity of pancreatectomy, patients may experience a prolonged postoperative recovery precluding timely administration of adjuvant chemotherapy; thus, preoperative therapy may enable use of systemic treatment for patients who would otherwise not receive it.

To our knowledge, there are currently no biomarkers for PDAC that influence clinical decision-making. However, emerging evidence from retrospective studies suggests circulating tumor DNA (ctDNA), a proxy for tumor burden, and keratin 17 (K17) expression in tumor cells, a validated biomarker of the most aggressive molecular subtype of PDAC, may have a role as prognostic biomarkers.^9,10,11,12,13,14^ We aimed to study whether dynamic changes in K17 and ctDNA expression were prognostic for resectable PDAC treated with perioperative mFOLFIRINOX in a prospective controlled trial. In the perioperative setting, these biomarkers may identify patients who will benefit from neoadjuvant mFOLFIRINOX, optimize treatment duration, and better predict relapse.

With the overall poor survival outcomes for resectable PDAC, we proposed to evaluate the efficacy of perioperative mFOLFIRINOX. At study initiation, adjuvant gemcitabine was the current standard of care, and we hypothesized that neoadjuvant chemotherapy would achieve earlier control of micrometastatic disease and increase survival compared with the adjuvant approach. Here, we present the results of the primary analysis of the Phase II Study of Peri-Operative Modified Folfirinox in Localized Pancreatic Cancer nonrandomized controlled trial for patients with resectable and selected borderline resectable PDAC.

## Methods

### Study Design and Participants

This study was a single-arm, open-label, phase 2 nonrandomized controlled trial conducted at the Yale Smilow Cancer Hospital. Eligible patients had untreated resectable PDAC. Resectability was defined as no evidence of tumor extension to the superior mesenteric artery, hepatic artery, celiac axis, aorta, or inferior vena cava and no evidence of occlusion or encasement of the superior mesenteric vein (SMV) or SMV/portal vein confluence by pancreatic protocol computed tomography and endoscopic ultrasonography. Patients underwent a multidisciplinary review and independent surgical oncology assessment to confirm resectability prior to enrollment. The study protocol is available in Supplement 1. Race was self-reported by study participants. The Yale University Institutional Review Board approved the protocol, and all patients provided written informed consent. The study was conducted in accordance with the Declaration of Helsinki and followed the Transparent Reporting of Evaluations With Nonrandomized Designs (TREND) reporting guideline.

### Procedures

Enrolled patients received 6 cycles of neoadjuvant mFOLFIRINOX followed by surgical resection and 6 adjuvant mFOLFIRINOX cycles. The Yale mFOLFIRINOX regimen consists of intravenous oxaliplatin, 85 mg/m^2^, followed by intravenous leucovorin, 400 mg/m^2^, intravenous irinotecan, 135 mg/m^2^, and intravenous 5-fluorouracil, 300 mg/m^2^, bolus then a continuous intravenous infusion of 5-fluorouracil, 2400 mg/m^2^, over 46 hours and pegfilgrastim on day 3.^15^ Assessments and specimen collections are provided in the eMethods in Supplement 2.

The ctDNA analysis was performed using a personalized, tumor-informed ctDNA assay (SignateraTM; Natera Inc). Whole-exome sequencing (WES) was performed on formalin-fixed paraffin-embedded tumor tissues (surgically resected) and matched normal blood. The WES data were used to design and develop individual patient-specific ctDNA assays by targeting a set of up to 16 somatic single-nucleotide variants found in the tumor.^16^ These assays were used to track the presence of ctDNA in patients’ blood plasma.

The K17 immunohistochemical detection was performed in diagnostic fine-needle aspirates (FNA) and biopsies and in surgical specimens after resection using the immunoperoxidase, and expression was scored by 2 independent surgical pathologists or cytopathologists (G. C.) using PathSQ scoring.^10,17^ K17 cutoff points, discriminating low and high values, were chosen using the lowest value of Akaike information criterion from a Cox proportional-hazard regression model. Aspirates and biopsies were excluded if the sample contained less than 100 tumor cells. Treatment response was scored using a modified Ryan Scheme.^17,18,19^ The WES was analyzed for Catalogue Of Somatic Mutations In Cancer (COSMIC) mutational signatures associated with impaired DNA repair.^20,21^

### Outcomes

The primary end point was 12-month progression-free survival (PFS) rate, defined as the proportion of patients alive without progression after 12 months. A PFS event was considered a clinical or radiographic progression or death. Patients who discontinued study treatments for alterative reasons were followed up for progression or death. Secondary end points included median overall survival (OS), median PFS, overall response rate by Response Evaluation Criteria in Solid Tumors (RECIST) version 1.1, and incidence of adverse events. Exploratory end points included the association of ctDNA, tumor genomics, and tumor K17 expression with survival.

### Statistical Analysis

Sample size estimate considered the primary end point of 12-month PFS as a proportion. Perioperative mFOLFIRINOX would be deemed worthy of further investigation if the observed 12-month PFS rate was statistically 50% or greater, which was considered to be the 12-month PFS rate with adjuvant gemcitabine.^22^ Considering the true 12-month PFS proportion under novel treatment regimen to be 0.66, 46 patients were necessary to achieve 81% power to conclude the 12-month PFS rate is indeed greater than 0.5 (rate under the null hypothesis) assuming a 1-sided alternative and α level of .10. More than 28 patients alive and progression free at 12 months were necessary to meet the primary end point goal. Primary and all secondary outcomes were analyzed according to the intention-to-treat principle. Survival curves were generated using Kaplan-Meier estimator and comparisons among subgroups were performed via log-rank test.^23^ All tests related to the secondary and exploratory analyses end points were 2-sided, and *P* values less than .05 were considered statistically significant. Descriptive statistics were used for safety and surgical outcomes analyses. Analyses were conducted using R version 4.1.2 (The R Foundation).

## Results

**Figure 1.**
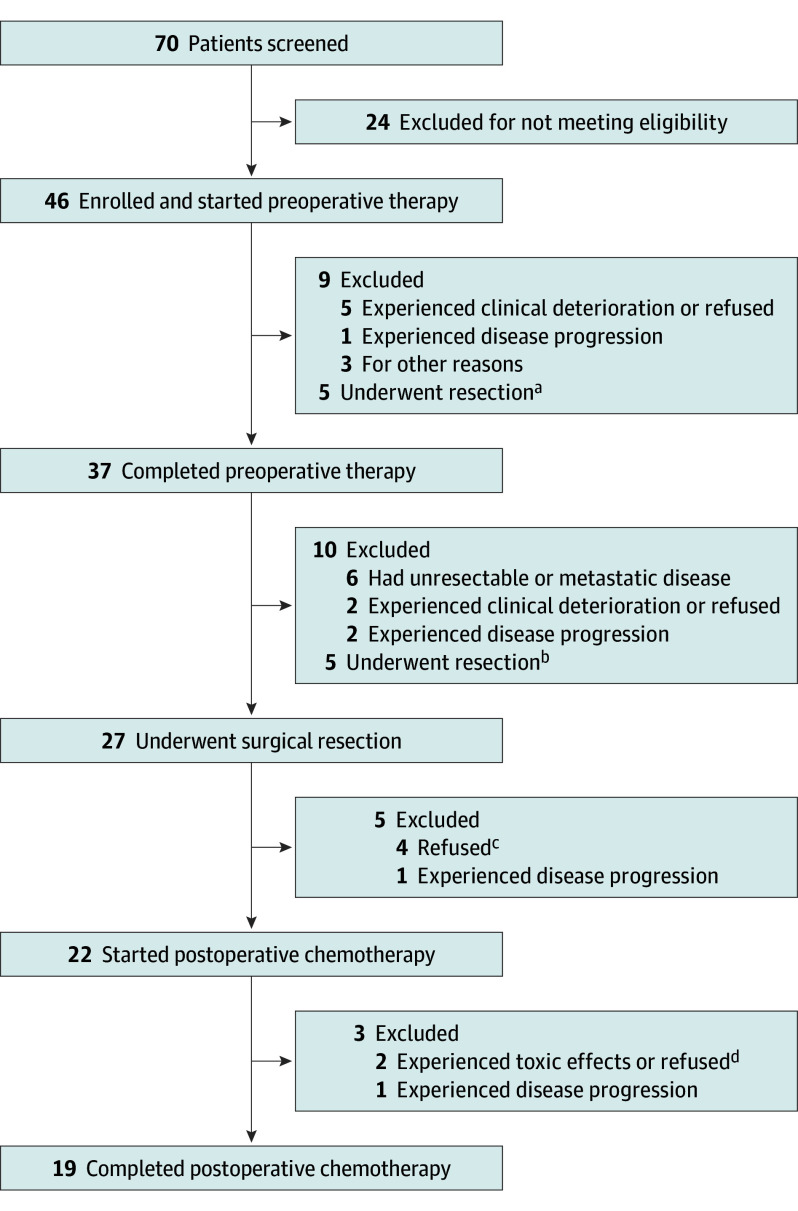
Trial Profile ^a^One patient discontinued modified 5-fluorouracil, leucovorin, oxaliplatin, and irinotecan (mFOLFIRINOX) to receive gemcitabine + nab-paclitaxel prior to surgery, 2 patients went to surgery after 1 cycle of mFOLFIRINOX, 1 patient went to surgery after 3 cycles of mFOLFIRINOX, and 1 patient underwent 12 total cycles of neoadjuvant mFOLFIRINOX and radiation prior to surgery. Three patients did not complete per-protocol therapy due to biliary tract complications, and all 3 ultimately underwent R0 surgical resection. ^b^After all 6 cycles of neoadjuvant mFOLFIRINOX; 2 patients received gemcitabine + nab-paclitaxel prior to surgery, 2 patients received additional mFOLFIRINOX, and 1 patient received additional mFOLFIRINOX, radiation, and gemcitabine + nab-paclitaxel prior to surgery. ^c^A total of 27 patients underwent surgery per protocol and 5 patients did not start adjuvant mFOLFIRINOX; 1 developed rapid progression, 1 pursued radiation for positive surgical margin, and 3 did not sufficiently recover from surgery to receive adjuvant therapy. ^d^Three patients prematurely discontinued adjuvant therapy, including 1 patient who discontinued mFOLFIRINOX after 1 cycle to pursue radiation for positive margin, 1 patient who discontinued to pursue adjuvant gemcitabine for improved tolerability, and 1 patient who progressed after 1 cycle of mFOLFIRINOX.

From April 3, 2014, to August 16, 2021, 46 patients were enrolled; 31 (67%) were male, and the median (range) age was 65 (46-80) years. Baseline patient characteristics are presented in the Table. At the time of data cut off (January 30, 2023), no patients were still receiving study treatment, and the median (range) follow-up was 63 (3-99) months. All enrolled patients started preoperative mFOLFIRINOX (Figure 1). No patients underwent staging laparoscopy. A total of 37 patients (80%) completed all 6 cycles of preoperative mFOLFIRINOX. A total of 33 patients (72%) underwent surgery, 6 (13%) had metastatic or unresectable disease identified intraoperatively, and 27 (59%) underwent successful surgical resection per protocol. Three patients discontinued study treatment prior to surgery for possible progression, 2 with non-RECIST local progression as assessed by the treating investigator and 1 with new lung metastases. A total of 10 additional patients (22%) underwent surgical resection off protocol (Figure 1). Thus, 37 patients underwent surgical resection.

**Table.** Baseline Characteristics

Characteristic	Patients, No. (%)
Total, No.	46
Age, median (range), y	65 (46-80)
Sex	
Female	15 (33)
Male	31 (67)
Self-reported race	
Black	5 (11)
White	40 (87)
Unknown race	1 (2)
ECOG performance status	
0	36 (78)
1	10 (22)
Location of primary tumor	
Pancreatic head	33 (72)
Pancreatic body/tail	13 (28)
Endobiliary stent	30 (65)
Histology	
Poorly differentiated	22 (48)
Moderately differentiated	17 (37)
Well differentiated	0
Unknown	7 (15)
Baseline CA 19-9, median (range), U/mL	131 (1-2445)
Vascular involvement by tumor	
No venous involvement	22 (48)
SMV or PV abutment without contour irregularity	16 (35)
SMV or PV abutment with contour irregularity	8 (17)
Genetic testing^a^	
BRCA2 germline mutation	1 (2)
No germline mutation	21 (46)
Unavailable	24 (52)

Abbreviations: ECOG, Eastern Cooperative Oncology Group; PV, portal vein; SMV, superior mesenteric vein.

^a^ A limited number of patients underwent genetic testing prior to 2018 when the National Comprehensive Cancer Network guidelines began recommending universal testing.

**Figure 2.**
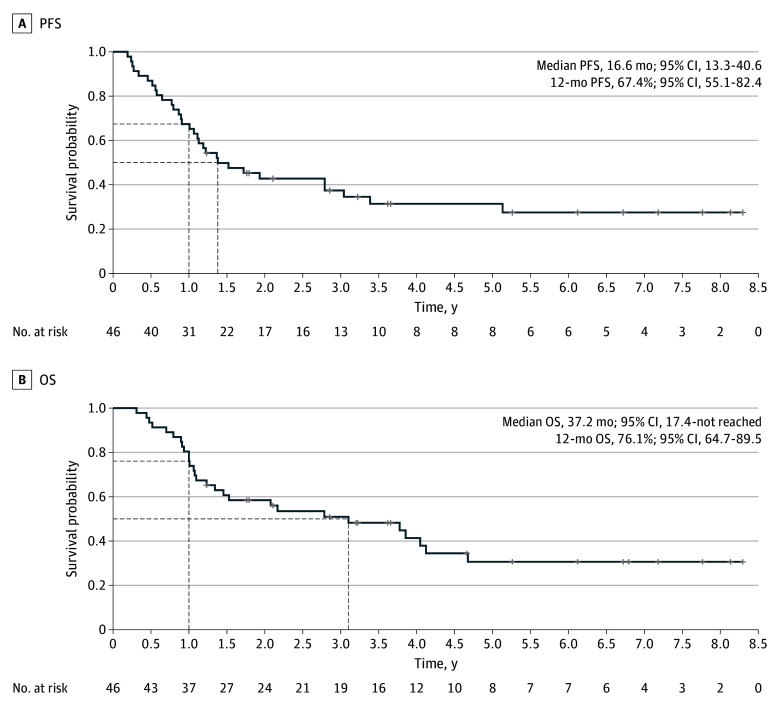
Survival Curves Progression-free survival (PFS) and overall survival (OS) curves for the intention-to-treat population.

The 12-month PFS rate for the intention-to-treat population was 67% (90% CI, 56.9-100) and was tested against the 1-sided alternative of .50 (*P* = .01) (Figure 2A). A total of 31 patients were alive and progression free at 12 months, and the Kaplan-Meier estimate of 12-month PFS rate was 67% (95% CI, 55.1-82.4) (Figure 2A). The median PFS and OS were 16.6 months (95% CI, 13.3-40.6) and 37.2 months (95% CI, 17.5-not reached), respectively (Figure 2). The 2-year OS was 59% (95% CI, 45.8-74.7). For 37 patients who completed all 6 cycles per-protocol preoperative mFOLFIRINOX, the median OS was 46.2 months (95% CI, 24.9-not reached); for 19 patients who received all 12 cycles of mFOLFIRINOX, the median OS was not reached (95% CI, 46.2-not reached). In patients with RECIST measurements, the overall response rate during neoadjuvant mFOLFIRINOX was 17% (6 of 37), and the disease control rate was 97% (eFigure 1 in Supplement 2). A total of 25 patients (93%) underwent R0 resection, while 2 patients (7%) had R1 resections. Pathologic response revealed 16 patients (59%) with poor or no treatment response after preoperative mFOLFIRINOX (eTable 1 in Supplement 2). The most frequently reported treatment-related adverse events are outlined in eTable 2 in Supplement 2. Preoperatively, 1 patient discontinued mFOLFIRINOX for treatment-related adverse events, and 1 or more dose reductions were required in 25 patients (54%).

**Figure 3.**
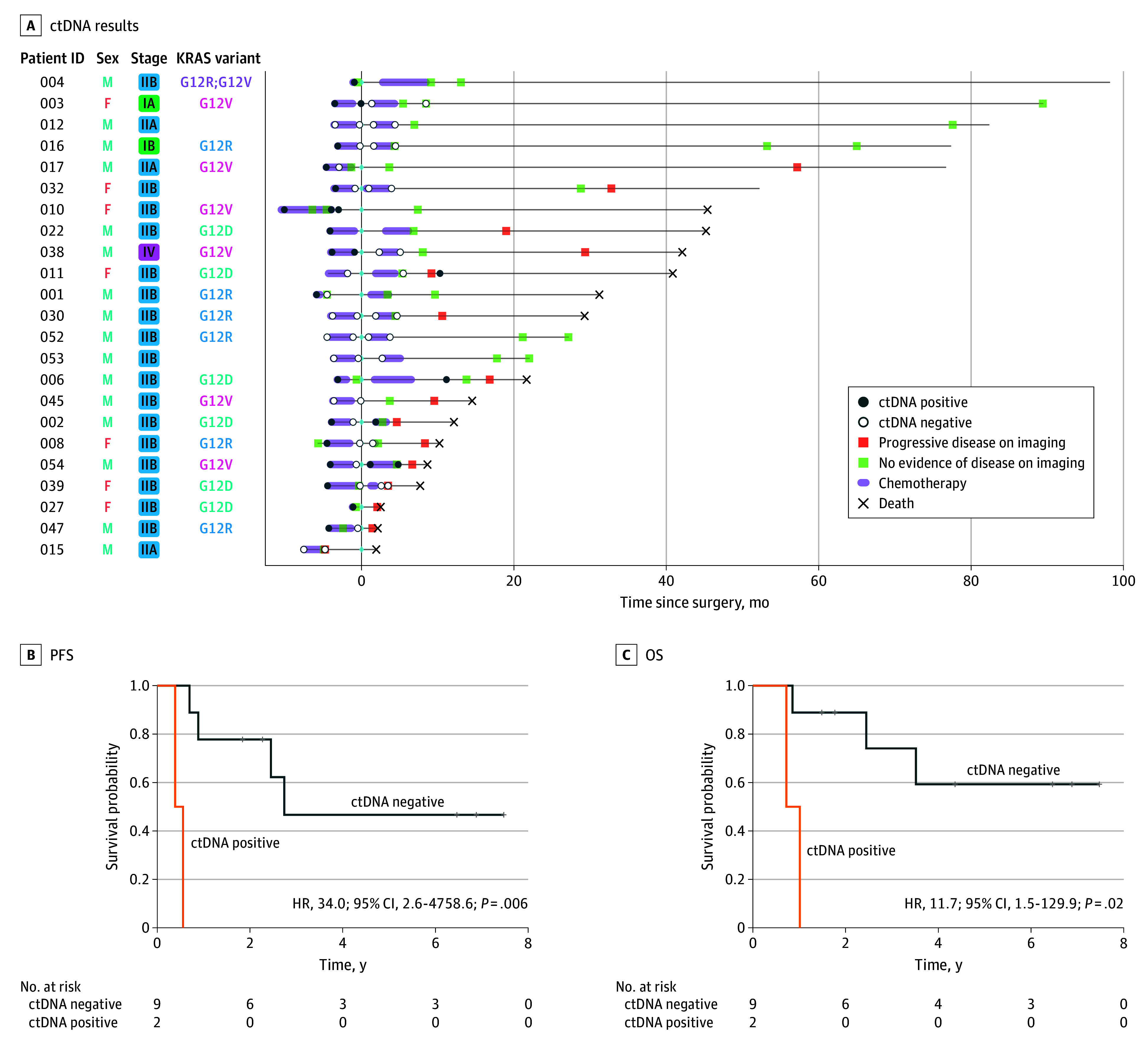
Circulating Tumor DNA (ctDNA) Results A, ctDNA results for patients with available results at 5 time points: baseline (pretreatment), preoperative (after 6 cycles of modified 5-fluorouracil, leucovorin, oxaliplatin, and irinotecan [mFOLFIRINOX]), postoperative (after surgical resection), end of treatment (after 6 cycles of adjuvant mFOLFIRINOX), and at the time of disease recurrence or progression. B, The progression-free survival for patients with detectable postoperative ctDNA levels. C, The overall survival for patients with detectable postoperative ctDNA levels. One patient who started postoperative FOLFIRINOX prior to postoperative ctDNA blood draw was excluded from postoperative ctDNA survival analysis. F indicates female; HR, hazard ratio; ID, identifier; M, male; OS, overall survival; PFS, progression-free survival.

Baseline ctDNA was detected in 16 of 22 patients (73%), and for 3 of 17 patients (18%), ctDNA remained positive after 6 cycles of mFOLFIRINOX (Figure 3). Postoperatively, ctDNA was detectable in 2 of 12 patients (17%), and after adjuvant mFOLFIRINOX, ctDNA was positive in 2 of 10 patients (20%) (Figure 3). There was no difference in median OS for patients with baseline undetectable ctDNA vs detectable ctDNA (hazard ratio [HR], 1.0; 95% CI, 0.3-3.8; *P* = .96). However, postoperative undetectable ctDNA demonstrated statistically significant improvement in PFS (HR, 34.0; 95% CI, 2.6-4758.6; *P* = .006) (Figure 3B) and OS (HR, 11.7; 95% CI, 1.5-129.9; *P* = .02) (Figure 3C) compared with the group with detectable ctDNA. One patient started postoperative mFOLFIRINOX prior to ctDNA collection and was excluded from the survival analysis. Furthermore, no statistically significant survival difference was identified for patients who converted from baseline ctDNA detected to undetected after mFOLFIRINOX compared with patients who continued to have detectable ctDNA (HR, 1.7; 95% CI, 0.3-8.5; *P* = .53).

**Figure 4.**
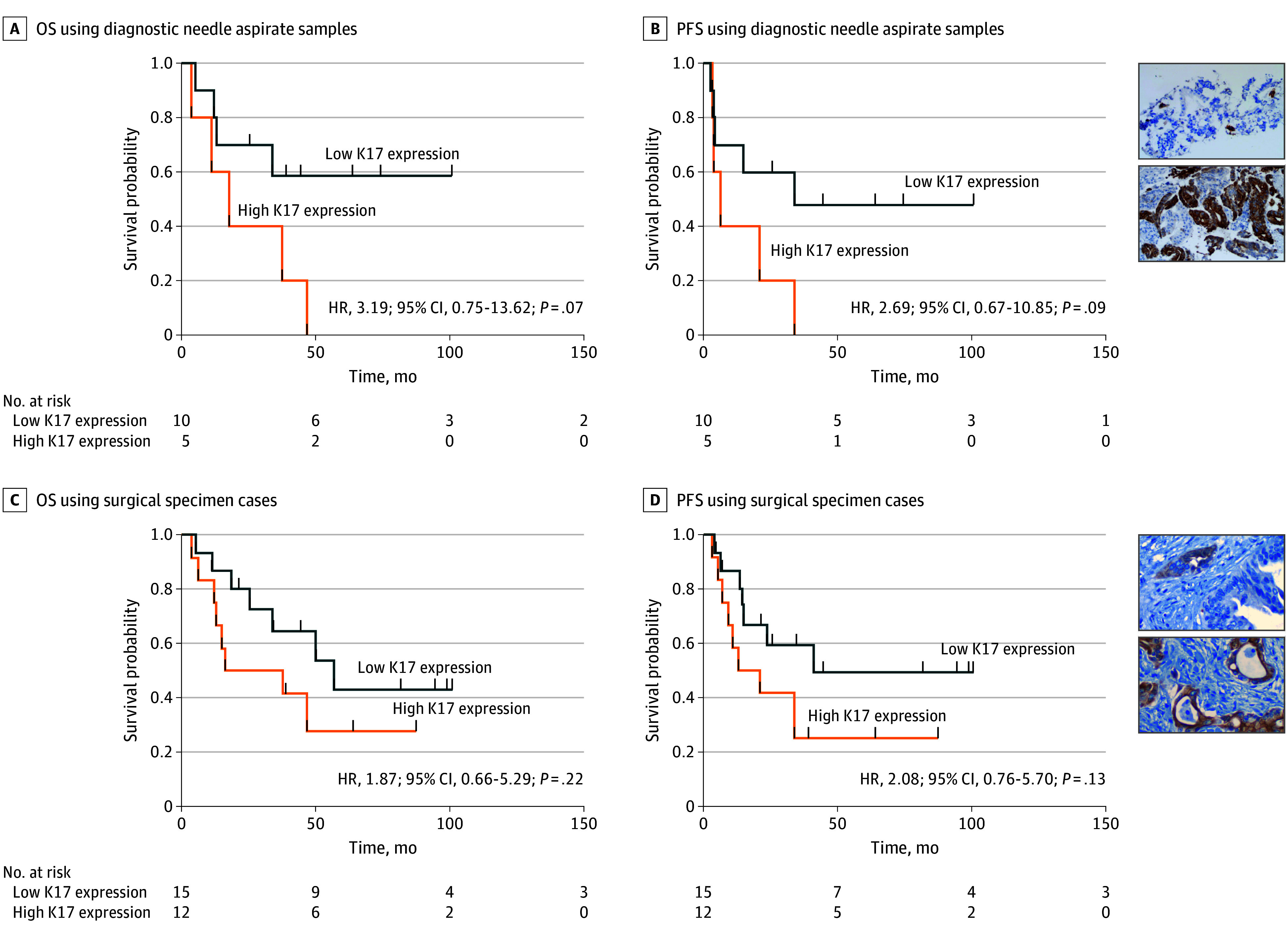
Keratin 17 (K17) Signatures A and B, Overall survival (OS) and progression-free survival (PFS) curves using diagnostic needle aspirate samples. C and D, Overall survival and progression-free survival curves using surgical specimen cases. Insets, Representative immunohistochemical stains for K17. HR indicates hazard ratio.

High K17 expression was identified in 5 of 15 tumors (33%) by FNA and 12 of 27 surgical specimens (44%). Although not statistically significant, high K17 expression was correlated with numerically shorter survival in FNAs for OS (HR, 3.2; 95% CI, 0.8-13.6; *P* = .07) and PFS (HR, 2.7; 95% CI, 0.7-10.9; *P* = .09) (Figure 4A and B; eFigure 2A in Supplement 2) and in surgical specimens for OS (HR, 1.9; 95% CI, 0.7-5.3; *P* = .22) and PFS (HR, 2.1; 95% CI, 0.8-5.7; *P* = .13) (Figure 4C and D; eFigure 2B and C in Supplement 2). Furthermore, K17 expression decreased in most tumors following neoadjuvant treatment (eFigure 2D in Supplement 2). Of note, mutational signature SBS15, which is associated with mismatch repair deficiency (dMMR), was correlated with decreased survival (eFigure 3 in Supplement 2). No patients had dMMR, and no other DNA repair signatures were prognostic.

## Discussion

In this study, perioperative mFOLFIRINOX was feasible and safe, with a high R0 resection rate, and this study met its primary objective, demonstrating a 12-month PFS rate of 67% (90% CI, 56.9-100) and a 2-year OS of 59% (95% CI, 45.8-74.7). Most patients completed 6 cycles of neoadjuvant therapy, and no patients had progression of the primary tumor by RECIST preoperatively. Furthermore, our results report, to our knowledge, the longest follow-up for any clinical trial using perioperative FOLFIRINOX for resectable PDAC and are the first to report the impact of tumor molecular features, K17, and tissue-informed ctDNA on survival. As outlined in Figure 1, multiple patients discontinued protocol therapy for reasons other than progression or adverse events. The challenges to complete per-protocol perioperative treatments reveal the limitations of PFS as an effective end point and highlight the superiority of OS for neoadjuvant or perioperative clinical trials with an aggressive malignant tumor, such as PDAC.

Our study was designed to compare to adjuvant gemcitabine survival, which was the standard of care in 2014.^24^ During our study, the standard of care evolved to adjuvant gemcitabine/capecitabine and ultimately to FOLFIRINOX.^5,25^ However, while long-term follow-up demonstrates that adjuvant FOLFIRINOX is superior to gemcitabine, the absolute difference between the arms is modest, with only a 7.1% improvement. Thus, the impact of early initiation of FOLFIRINOX prior to surgery is a highly relevant question. To our knowledge, the SWOG 1505 trial^8^ was the first study to report the use of perioperative FOLFIRINOX for resectable PDAC, which evaluated FOLFIRINOX in arm 1 and gemcitabine/nab-paclitaxel in arm 2. The trial revealed the feasibility of preoperative FOLFIRINOX, with 84% of patients completing preoperative treatment, but neither arm met the prespecified primary end point for 2-year OS of 58% or greater.^26^ The feasibility of neoadjuvant FOLFIRINOX for borderline resectable PDAC followed by chemoradiotherapy has also been shown with a completion rate of 79%, a 2-year PFS of 43%, and median OS of 37.7 months.^27^ In contrast, the preliminary results of the phase 2 NORPACT-1 randomized clinical trial revealed a nonstatistically significant trend toward decreased OS with perioperative FOLFIRINOX compared with adjuvant FOLFIRINOX.^28^ However, adherence to both preoperative and postoperative FOLFIRINOX was poor, with definitive conclusions about FOLFIRINOX sequencing remaining an open question. Collectively, these experiences highlight the difficulties with interpreting data from small perioperative PDAC clinical trials and the importance of the ongoing phase 3 Alliance randomized clinical trial.^29^

While it may seem reasonable to use a survival rate with adjuvant FOLFIRINOX as a historical control when assessing neoadjuvant strategies, this comparison is invalid, as it does not account for the inherent differences in the patient populations enrolled to neoadjuvant and adjuvant studies. For example, patients enrolled in adjuvant studies have undergone successful and uncomplicated surgery without findings of occult metastatic disease or early recurrence, which would preclude enrollment. In contrast, in neoadjuvant studies, enrollment is essentially at diagnosis, which includes patients with aggressive tumor biology, occult advanced disease, and patients who experience unexpected surgical complications precluding adjuvant treatment. These systematic differences in patient enrollment need to be acknowledged when designing, powering, and interpreting nonrandomized perioperative studies for PDAC.

Previous observations support the prognostic role of ctDNA in resectable PDAC at baseline and in the postoperative setting.^14^ To our knowledge, our findings in collaboration with Natera are the first to evaluate the prognostic significance of ctDNA testing with a clinically validated, personalized, tumor-informed assay (SignateraTM) in patients with resectable PDAC with perioperative mFOLFIRINOX. Here, we demonstrated postoperative detectable ctDNA levels to be highly prognostic. The Signatera test is tissue informed, and in our trial, only patients who underwent successful resection and had adequate tissue were amenable to testing. This is particularly relevant in patients treated in the neoadjuvant setting, as those with major pathologic responses had insufficient tissue for ctDNA testing. Moreover, the reliability of ctDNA results before FOLFIRINOX use when using a test from a post-FOLFIRINOX tumor specimen is uncertain. For example, nontruncal mutations may increase in posttreatment tumors, which needs to be accounted for when running the ctDNA test. In addition, we did not observe statistically significant survival differences for patients who converted from baseline detectable ctDNA to undetectable after 6 cycles of mFOLFIRINOX vs patients who had persistently detectable ctDNA. Thus, preoperative ctDNA detection after induction mFOLFIRINOX may not guarantee relapse and should not prevent a potentially curative operation.

Tumor K17 expression has emerged as a defining biomarker of the most aggressive forms of PDAC, and it is currently being tested as a biomarker to predict chemotherapy response in this and other phase 2 clinical trials.^9,10,11,12,13,30,31^ Here, we report that K17 was associated with numerically decreased survival whether detected at diagnosis in FNA or in the postneoadjuvant setting using surgical specimens, although these exploratory observations were not statistically significant and require further validation. An additional exploratory subset analysis of the ctDNA cohort revealed that high K17 expression may stratify survival of patients with poor response (median OS less than 25 months), although this was not statistically significant (eFigure 2E in Supplement 2). Moreover, high K17 expression further stratified OS for detectable ctDNA (eFigure 2F in Supplement 2). Thus, by interaction analyses, we identified that K17 expression may enhance survival stratification with clinical response and ctDNA status. To our knowledge, this study is the first to suggest that molecular subtyping by rapid and immunohistochemical-based tests in both diagnostic aspirates and in resected tumors after neoadjuvant mFOLFIRINOX may provide patient survival information not provided by other clinical or prognostic biomarkers.

Mutational signature analysis of WES revealed signature SBS15, which is associated with dMMR, to be associated with a worse prognosis, and no signatures of homologous recombination deficiency were identified. The chemoresistance of dMMR tumors is one possible explanation why SBS15 was associated with decreased survival.^32,33,34^ Accounting for overlap in germline WES and standard-of-care genetic testing (Table), we identified a germline deleterious homologous recombination deficiency alteration (*BRCA2*) in a patient with more than 5 years of OS. Descriptive analysis of mutational signatures suggests genomic differences may be associated with ctDNA status (eFigure 4 in Supplement 2) and may vary by OS subgroup: those with poor (less than 25 months), intermediate (25 to 49 months), and exceptional (50 months or more) OS (eFigure 5 in Supplement 2).

### Limitations

This study has limitations. One limitation is our definition of resectability. Patients with SMV and/or portal vein contour irregularity, which by current National Comprehensive Cancer Network guidelines would be considered borderline resectable, were included. This limitation may have negatively impacted our efficacy results by allowing enrollment of patients with more advanced disease that may require more extensive or complex surgery. Multidisciplinary review of pancreatic protocol computed tomography minimizes the impact of this limitation, and only 8 patients had venous contour irregularity (Table). Furthermore, WES signature analysis is suboptimal without supporting RNA sequencing data. Our study also had a considerable dropout rate, with multiple patients discontinuing per-protocol treatment to pursue alternate approaches, such as early surgical resection, gemcitabine/nab-paclitaxel, and radiation, despite the absence of progression or toxic effects necessitating treatment discontinuation (Figure 1).

## Conclusions

In conclusion, the Phase II Study of Peri-Operative Modified Folfirinox in Localized Pancreatic Cancer nonrandomized controlled trial met its primary end point with a 12-month PFS rate of 67%, and we also report exceptional survival with high R0 resection rates for patients who completed per-protocol therapy. Our exploratory analysis suggests that ctDNA and K17 are promising biomarkers for PDAC treated with perioperative mFOLFIRINOX. However, future studies are needed to determine the role of ctDNA and K17 to enhance patient selection, predict patient outcomes, and optimize timing of surgery. We also demonstrate the feasibility of 6 cycles of perioperative mFOLFIRINOX for resectable PDAC. Thus, while our reported survival rates are promising, a randomized clinical trial is critical to determine whether perioperative mFOLFIRINOX enhances cure rates compared with adjuvant FOLFIRINOX.

## Notes

Supplement 1. Trial Protocol

Supplement 2.
eMethods.
**eFigure 1.** Waterfall Plot **eTable 1.** Surgical Outcomes **eFigure 2.** Supplementary Keratin 17 Signatures **eFigure 3.** Mutational Signatures for All Enrolled Patients **eTable 2.** Frequency of Treatment Emergent Adverse Events and Laboratory Abnormalities With ≥10% Incidence **eFigure 4.** Mutational Signatures for Baseline ctDNA-Negative and ctDNA-Positive Cohorts **eFigure 5.** Mutational Signatures for Exceptional, Intermediate, and Poor Responders

Supplement 3. Data Sharing Statement
