# Supplementary material for: Perioperative Modified FOLFIRINOX for Resectable Pancreatic Cancer: A Nonrandomized Controlled Trial
Source: JAMA Oncol. 2024 Jun 20;10(8):1027–35. doi: 10.1001/jamaoncol.2024.1575 (PMC11190830; doi:10.1001/jamaoncol.2024.1575)
Supplement: Supplement 1. — Trial Protocol [file jamaoncol-e241575-s001.pdf]

Supplementary Online Content

This supplement contains the full clinical trial protocol.

TITLE: PHASE II STUDY OF PERI-OPERATIVE MODIFIED FOLFIRINOX IN LOCALIZED PANCREATIC CANCER

HIC Protocol Number:	1306012255
Version Number:	8.0
Version Date:	12-Sep-2018
Sponsor:	Yale Comprehensive Cancer Center
Principal Investigator:	Jill Lacy, M.D.<br>Yale University School of Medicine<br>333 Cedar Street, 219 WWW<br>PO Box 208032<br>New Haven, CT 06520-8032<br>Ph: 203-737-1600; Fax: 203-785-7531<br>Jill.lacy@yale.edu
Statistician:	Daniel Zelterman, PhD
Study Coordinator:	Christina Wiess
Data Manager:	Kamile Pamorada
Study Medications:	Leucovorin, 5-fluorouracil, irinotecan, oxaliplatin
Financial Support:	Departmental

TABLE OF CONTENTS

1.0 BACKGROUND/RATIONALE... 3
2.0 OBJECTIVES... 7
3.0 STUDY DESIGN... 8
4.0 ON-STUDY GUIDELINES AND SAFETY PLAN........................... . 9
5.0 ELIGIBILITY CRITERIA.. 10
6.0 TREATMENT PLAN... 12
7.0 STUDY DRUGS/DOSE MODIFICATIONS/TOXICITY MANAGEMENT.. 13
7.1 DOSES/SCHEDULE OF STUDY DRUGS.............................. 13
7.2 DRUGS: FORMULATION/ADMINISTRATION/TOXICITY........... 14
7.3 DOSE MODIFICATIONS AND TOXICITY MANAGEMENT......... 21
8.0 REQUIRED CLINICAL AND LABORATORY EVALUATIONS............... 26
9.0 CRITERIA FOR RESPONSE AND PROGRESSION.......................... 28
10.0 SUBJECT DISCONTINUATION.. 33
11.0 STUDY DISCONTINUATION.. 34
12.0 STATISTICAL METHODS.. 34
13.0 ADVERSE EVENT ANALYSIS, DEFINITION AND REPORTING.......... 35
14.0 YALE PRINCIPAL INVESTIGATOR SAE REPORTING REQUIREMENTS.. 38
15.0 YALE SAFETY REPORTING AND MONITORING (DSMP)................. 39
16.0 RETENTION OF RECORDS... 40
17.0 RESEARCH ETHICS AND HUMAN SUBJECT PROTECTION............ 40
18.0 REFERENCES... 42
APPENDIX A: STANDARD PROCEDURE FOR COLLECTION AND STORAGE OF PLASMA AND BUFFY COAT SPECIMENS 45

1.0 BACKGROUND AND RATIONALE

1.1 BACKGROUND

Pancreatic cancer is the fourth leading cause of cancer-related death in men and women. There were an estimated 43,140 new cases and 36,800 deaths from pancreatic cancer in United States in 2010, and the five year survival for all patients with pancreatic adenocarcinoma is <5% (1).

While surgical resection (pancreaticoduodenectomy or distal pancreatectomy) is considered the only potentially curative treatment, only 20-25% of patients present with potentially resectable disease. Among patients who are able to undergo surgical resection, the 5 year survival rate is just 4-21% due to the high rate of metastatic and local-regional recurrence (2-4). These disappointing results are likely attributable to early hematogenous dissemination such that sub-clinical metastastases are present at the time of diagnosis in most patients, as well as positive margins at the time of resection. Patients with resectable disease who have positive margins (R1 or R2 resection) have survival rates comparable to patients with locally advanced unresectable disease (5-7).

At present, the standard of care for resectable pancreatic cancer supports the use of adjuvant chemotherapy with gemcitabine or leucovorin-modulated 5-fluorouracil (5-FU) for 6 months. As reported in the CONKO-001 trial, the use of adjuvant gemcitabine for resectable pancreatic adenocarcinoma resulted in a significant improvement in disease-free survival compared to observation (13.4 months versus 6.9 months) (2). However, gemcitabine was associated with only a modest improvement in survival (median overall survival 22.8 months versus 20.2 months; five-year survival 21% versus 9%) (8). In the ESPAC-1 trial, both the two-year (40 versus 30 percent) and five-year (21 versus 8 percent) survival rates were significantly greater among patients randomized to postoperative bolus leucovorin-modulated 5-FU for six months compared to those who did not receive it (3). The median survival for patients treated with 5-FU versus no chemotherapy was 20.1 versus 15.5 months, respectively. Subsequently, in the ESPAC-3 trial, adjuvant gemcitabine was compared to 5-FU (4). These regimens were shown to be virtually identical in terms of efficacy (median survival 23.6 mo versus 23

mo), although 5-FU was associated with more stomatitis and diarrhea. Thus, the standard of care for patients who have undergone definitive resection of localized pancreatic cancer is the administration of six months of adjuvant gemcitabine, although 5-FU is an acceptable alternative.

Although gemcitabine or bolus 5-FU remain the standard of care in the adjuvant setting, the most effective regimen for metastatic pancreatic adenocarcinoma is the combination regimen FOLFIRINOX (oxaliplatin 85 mg/m^2 over 2 hrs, followed by irinotecan 180 mg/m^2 over 90 min and leucovorin 400 mg/m^2 over 2 hrs, followed by 5-fluorouracil 400 mg/m^2 bolus and 2,400 mg/m^2 46h continuous infusion). FOLFIRINOX was compared to gemcitabine in a randomized phase Ill trial in patients with metastatic pancreatic cancer (9). This study showed significant improvements in overall survival (11.1 v 6.8 mo), progression free survival (6.4 v 3.3 mo) and response rate (31.6% v 9.4%) with FOLFIRINOX compared to gemcitabine. FOLFIRINOX was associated with significantly more grade 3/4 toxicity than gemcitabine (neutropenia, 45.7% v 21.0%; febrile neutropenia, 5.4% v 1.2%; fatigue, 23.6% v 17.8%; vomiting, 14.5% v 8.3%; diarrhea, 12.7% v 1.8%). There was one toxic death in each arm. Based on this study, FOLFIRINOX is considered the most effective regimen for metastatic pancreatic cancer, and it has emerged as the new standard of care in appropriate patients with a good performance status.

Given concerns regarding excessive toxicity of FOLFIRINOX, most practitioners are reluctant to use full doses of all three chemotherapeutic agents. A retrospective review of all patients treated at Smilow Cancer Center between June 2010 and June 2011 with FOLFIRINOX demonstrated that routine modest dose attenuations in combination with pegfilgrastim support was associated with improved tolerability and comparable efficacy compared to FOLFIRINOX-treated patients reported by Conroy et al (10,11). In this retrospective review, 35 patients were treated (16 locally advanced unresectable; 19 metastatic). 29 patients received dose attenuations with the first cycle. Median relative doses of irinotecan and bolus fluorouracil were less than those reported by Conroy (64% v 81% and 66% v 82%, respectively). Response rate was 47% and did not differ significantly from the response rate of 32% reported by Conroy ($p = 0.19$). Overall survival (in the metastatic cohort) at 6 and 12 months was comparable to OS reported by Conroy. The incidences of grade 3/4 toxicities (febrile neutropenia, anemia, thrombocytopenia, diarrhea, vomiting, fatigue [$p=0.009$], neutropenia [$p<0.0001$] were less than reported by Conroy et al, and we observed no treatment-related deaths.

Based on this experience, dose-attenuated FOLFIRINOX (25% reduction in dose of irinotecan and bolus 5-FU), with routine use of pegfilgrastim, is being prospectively evaluated in patients with advanced pancreatic cancer (locally advanced unresectable or metastatic) in a Yale-sponsored multi-institutional Phase II trial. Although this study is ongoing, preliminary analysis of 27 patients validates the tolerability and safety of this regimen. Of note, at this institution, 6 patients with locally advanced pancreatic cancer have undergone successful surgical resection within one month of completion of 4 or more cycles of FOLFIRINOX. In this small cohort of patients who received neoadjuvant FOLFIRINOX for locally advanced disease, we have observed no delays in moving to surgery and no unusual or unexpected post-operative complications in the post-operative period.

While FOLFIRINOX is clearly superior to gemcitabine in the metastatic setting, there are no data regarding its efficacy and tolerability in the neoadjuvant or adjuvant setting in patients with resectable disease. Given the significant improvement in overall survival and progression free survival in the metastatic setting, the use of FOLFIRINOX in the neoadjuvant and/or adjuvant setting in patients with resectable pancreatic cancer may lead to substantial improvements in PFS and OS compared to adjuvant gemcitabine.

There are several potential advantages in administering systemic chemotherapy pre-operatively in patients with resectable pancreatic cancer, as follows: (1) early systemic therapy of micrometastatic disease may be more effective than delayed systemic therapy after surgery, due to lower burden of occult metastatic disease; (2) the cytoreductive effect of pre-operative chemotherapy may increase the rate of margin-negative RO resections (v R1 or R2); (3) adjuvant chemotherapy administration after pancreatectomy is often delayed for months due to post-operative recovery, potentially reducing efficacy; (4) 25% of patients never receive adjuvant chemotherapy due to delayed post-operative recovery (12); (5) the administration of pre-operative chemotherapy will identify patients with rapidly progressive disease who will not benefit from surgery.

Potential disadvantages of neoadjuvant chemotherapy include the requirement for biliary decompression before chemotherapy and potential for complications associated with biliary stents, and delayed surgery allowing progression locally to a nonresectable stage in patients whose disease did not respond to chemotherapy. Despite these theoretical disadvantages, multiple small phase II studies of neoadjuvant therapies with

gemcitabine- or 5-FU-based regimens, usually with concurrent radiotherapy, have demonstrated the safety and feasibility of this approach in patients with resectable or borderline resectable disease (13-20). In general, patients who undergo neoadjuvant therapies have not experienced increased pre- and peri-operative complications, and rates of hepatic toxicity and biliary stent-related complications are low. Moreover, these studies demonstrated a negligible rate of local-only progression precluding surgery. For example, in a randomized phase 2 trial of neoadjuvant chemotherapy in resectable pancreatic cancer with gemcitabine alone versus gemcitabine combined with cisplatin, only one of 50 patients was unable to proceed to surgery due to local progression (16).

While median survival durations from some of these small uncontrolled trials of neoadjuvant therapy for localized pancreatic cancer compare favorably to those reported with adjuvant therapy approaches, whether preoperative therapy is better than postoperative therapy remains unknown, as there are no randomized trials comparing the two approaches. Notably, none of the published studies of neoadjuvant therapy have incorporated FOLFIRINOX. At present, neoadjuvant therapy for resectable pancreatic cancer is not considered standard of care.

Based on the superior efficacy of FOLFIRINOX compared to gemcitabine in metastatic pancreatic cancer, and the potential advantages of neoadjuvant chemotherapy, we propose to evaluate the efficacy of peri-operative (neoadjuvant and adjuvant) FOLFIRINOX in patients with resectable pancreatic adenocarcinoma.

1.2 STUDY RATIONALE AND HYPOTHESIS

In summary, patients with localized resectable pancreatic adenocarcinoma have a 5 year survival rate of 4-21% after surgical resection (pancreaticoduodenectomy or distal pancreatectomy). Although the use of adjuvant gemcitabine or bolus 5-FU is the current standard of care, these regimens have only a modest impact on overall survival compared to observation. FOLFIRINOX has been shown to significantly increase survival, progression-free survival, and response rate compared to gemcitabine in metastatic pancreatic cancer. Thus, it is likely that FOLFIRINOX will improve outcomes compared to gemcitabine in patients with resectable pancreatic cancer. Based on the potential advantages of administration of systemic therapy pre-operatively in patients with resectable pancreatic cancer, we propose the utilization of peri-operative (neoadjuvant and adjuvant) FOLFIRINOX. Finally, since modest dose reductions of

bolus 5-FU and irinotecan in patients with advanced pancreatic cancer may be associated with improved tolerability and similar efficacy compared to full dose FOLFIRINOX, we will utilize dose-attenuated modification of FOLFIRINOX, or mFOLFIRINOX, with a 25% reduction in the doses of bolus 5-FU and irinotecan.

We will evaluate peri-operative (neoadjuvant and adjuvant) mFOLFIRINOX in patients with resectable pancreatic adenocarcinoma. We hypothesize that the use of peri-operative mFOLFIRINOX will be associated with improved PFS compared to historical controls treated with adjuvant gemcitabine.

2.0 OBJECTIVES

2.1 PRIMARY

The primary objective of this study is to determine the progression-free survival in patients with resectable non-metastatic pancreatic cancer treated with peri-operative mFOLFIRINOX.

2.2 SECONDARY

1) Determine overall survival
2) Determine objective response rate after neoadjuvant mFOLFIRINOX

2.3 EXPLORATORY

1) Compare RO resection rate and pathologic stage with institutional historical controls who did not receive neoadjuvant therapy
2) Correlate early metabolic response, determined by changes in glucose metabolism using PET scanning, with pathologic response, RO resection, and pathologic stage.
3) Correlate early metabolic response, determined by changes in glucose metabolism using PET scanning, with progression-free and overall survival
4) Correlate pre-operative response of CA19-9 with progression-free and overall survival
5) Collect and bank serial serum and plasma specimens from subjects for future correlative biomarker studies.
6) Collect and bank tumor tissue from subjects prior to treatment (from the diagnostic EUS-guided biopsy) and after treatment with six cycles of FOLFIRINOX (from the surgical specimen) for future correlative biomarker studies.

APPROVED BY THE YALE UNIVERSITY IRB 8/7/2020

3.0 STUDY DESIGN

3.1 DESCRIPTION OF STUDY

This is a Phase 11 open-label study to determine the anti-tumor efficacy of peri-operative (neoadjuvant and adjuvant) mFOLFIRINOX in patients with resectable pancreatic adenocarcinoma. Patients will receive 6 cycles of mFOLFIRINOX every 2 weeks as follows: Oxaliplatin 85 mg/m^{2}, followed by folinic acid 400 mg/m2 infused over 120 minutes and irinotecan 135 mg/m^{2} infused over 90 minutes, followed by 5-fluorouracil 300 mg/m^{2} IV bolus, followed by 2,400 mg/m^{2} continuous infusion for 46 hours. Levoleucovorin may be substituted for folinic acid at a dose of 200 mg/m2 infused over 120 minutes.

After the initial 6 cycles of mFOLFIRINOX, patients will undergo re-evaluation for resection with cross-sectional imaging (CAT scan or MRI scan); EUS is optional if deemed necessary to assess resectability. Patients who are resectable will undergo surgical resection between 3 and 8 weeks after completion of neoadjuvant mFOLFIRINOX. Post-operatively, patients will receive an additional 6 cycles of mFOLIFIRINOX. Patients will be eligible to start the adjuvant treatment as part of the study for up to 12 weeks after surgery.

Patients who complete the study treatment phase without disease progression will enter a post-study surveillance phase. They will undergo history, physical exam, laboratory assessments at two month intervals and cross-sectional imaging at 4 months intervals for three years, and then at six month intervals for two additional years. After five years, patients will be followed annually until death.

3.2 RATIONALE FOR STUDY DESIGN

Since FOLFIRINOX has been shown to significantly increase progression-free and overall survival compared to gemcitabine in patients with metastatic pancreatic cancer, there is compelling rationale for utilizing FOLFIRINOX rather than gemcitabine in patients with resectable pancreatic cancer. Our retrospective study and on-going prospective phase II study at Yale have shown improved tolerability and comparable efficacy of mFOLFIRINOX compared to FOLFIRINOX as reported by Conroy et al.

Therefore, we propose an open label phase II study to evaluate the efficacy of peri-operative (neoadjuvant and adjuvant) mFOLFIRINOX in patients with localized resectable pancreatic cancer. We hypothesize that the use of mFOLFIRINOX will be associated with a significant improvement in PFS when compared to historical controls treated with gemcitabine.

3.3 OUTCOME MEASURES

3.3.1 PRIMARY OUTCOME MEASURES

The primary outcome measure of this study is progression-free survival of patients with resectable non-metastatic pancreatic cancer treated with modified FOLFIRINOX in the neoadjuvant and adjuvant settings.

3.3.2 SECONDARY OUTCOME MEASURES

The secondary outcome measures are:
1) Overall survival
2) Objective response rate after neoadjuvant mFOLFIRINOX

3.4 EXPECTED ACCRUAL

Expected accrual is 46 patients over five years (see Section 12, Statistical Methods). We anticipate accrual will be completed in five years, based on the number of pancreatic resections for adenocarcinoma that are performed annual at Yale New Haven Hospital annually (>40 per year).

4.0 ON-STUDY GUIDELINES AND SAFETY PLAN

4.1 GENERAL ON-STUDY GUIDELINES

This clinical trial can fulfill its objectives only if patients appropriate for this trial are enrolled. All relevant medical and other considerations should be taken into account when deciding whether this protocol is appropriate for a particular patient. Physicians should consider the risks and benefits of any therapy, and therefore only enroll patients for whom this treatment is appropriate. Although they may not be considered as formal

subject selection criteria, as part of this decision making process, physicians should recognize that the following may seriously increase the risk to the patient entering this protocol:

- Psychiatric illness that would prevent the patient from giving informed consent.
- Patient is not deemed a candidate for mFOLFIRINOX based on overall condition and co-morbidities.
- A medical condition such as active/uncontrolled infection or cardiac disease that would make this protocol unreasonably hazardous for the patient in the opinion of the treating physician.

4.2 mFOLFIRINOX-SPECIFIC SAFETY GUIDELINES

See Section 7 for detailed instructions for safety guidelines and management of FOLFIRINOX-related toxicities.

5.0 ELIGIBILITY CRITERIA

The eligibility criteria for this study are as follows.

Disease Characteristics:

- Pathologic or cytologic documentation of pancreatic adenocarcinoma
- Resectable pancreatic adenocarcinoma disease as defined as follows:

  -No evidence of extrapancreatic disease by cross sectional imaging, PET scan, or laparoscopy, including nodal involvement beyond the peripancreatic tissues and/or distant metastases;
  -No evidence of tumor extension to superior mesenteric artery, hepatic artery, celiac axis, aorta, or inferior vena cava, and no evidence of occlusion or encasement of the superior mesenteric vein or superior mesenteric vein/portal vein confluence, as assessed by CT using pancreatic protocol (or MRI in patients who cannot undergo CT) and EUS

Prior Therapy:

- No prior treatment (chemotherapy, biological therapy, or radiotherapy) for resectable pancreatic cancer
- No prior treatment with oxaliplatin, irinotecan, fluoruouracil or capecitabine
- Patients who received chemotherapy >5 years ago for malignancies other than pancreatic cancer are eligible
- There is no evidence of the second malignancy at the time of study entry
- >4 weeks since major surgery
- No other concurrent anticancer therapy

Patient Characteristics:

- ECOG Performance Status: 0-1
- Age 18
- No other malignancy within past five years except basal cell carcinoma of the skin, cervical carcinoma in situ, or nonmetastatic prostate cancer
- Paraffin block or slides must be available
- Adequate organ function
- No interstitial pneumonia or extensive and symptomatic interstitial fibrosis of the lung
- No grade 2 sensory peripheral neuropathy
- No uncontrolled seizure disorder, active neurological disease, or known CNS disease
- No significant cardiac disease, including the following: unstable angina, New York Heart Association class II-IV congestive heart failure, myocardial infarction within six months prior to study enrollment
- No history of chronic diarrhea
- Not pregnant and not nursing
- No other medical condition or reason that, in the opinion of the investigator, would preclude study participation
- Laboratory parameters as follows: absolute neutrophil count 21,500/uL, platelet count 2100,000/uL, hemoglobin 29 g,/dL, creatinine <1.5 X ULN or estimated GFR >30 ml/min, bilirubin _:::1.5 X ULN, AST and ALT _:::3 X ULN, negative pregnancy test in women of childbearing age

6.0 TREATMENT PLAN

This is an open-label non-randomized Phase II study. Patients will receive neoadjuvant treatment with mFOLFIRINOX every two weeks (initiated on day 1 of every 2 week cycle). Therapy will be administered in an outpatient setting under medical supervision. Protocol treatment is to begin within 14 days of registration. One cycle will be defined as 14 days or two weeks of treatment.

Prior to surgery, patients will receive 6 cycles (12 weeks) of neoadjuvant mFOLFIRINOX. An interval response assessment PET scan will be performed after 3 cycles, and if there is no radiographic evidence of disease progression, patients will receive 3 additional cycles of mFOLFIRINOX. After completion of 6 cycles, patients will undergo re-assessment for resection, including a pre-operative CT scan using pancreatic protocol (or MRI scan in patients who cannot undergo CT scan) and surgical evaluation; EUS and PET scan are optional if deemed necessary to assess resectability. Patients who are deemed surgical candidates after re-assessment will undergo a pancreaticoduodenectomy or distal pancreatectomy within 3-8 weeks of completion of 6 cycles of neoadjuvant mFOLFIRINOX.

Full pathologic evaluation and staging will be performed on resected disease. Patients will continue to be seen every 2 weeks in the medical oncology setting to assess readiness to start adjuvant therapy.

After surgery, patients will receive 6 cycles of adjuvant mFOLFIRINOX. Patients will start adjuvant therapy with mFOLFIRINOX after they have returned to ECOG performance status of 0-1, have no open incisions, have normalization of labs and have demonstrated stable or increasing weight for >1 week. Patients will be eligible to receive adjuvant treatment as part of the study if treatment is initiated within 12 weeks after surgery.

Chemotherapy will consist of mFOLFIRINOX administered every two weeks. The details of administration of each drug in mFOLFIRINOX are described in section 7. Treatment is initiated on day 1 of a two week cycle. The doses of drugs and sequence of administration are as follows:

- Oxaliplatin 85 mg/m^2 IV infused over two hours, followed by

- Leucovorin 400 mg/m^2 IV (or levoleucovorin 200 mg/m^2 IV) over two hours

- Irinotecan 135 mg/m^2 IV over 90 minutes (concurrent with leucovorin during the last 90 min of the leucovorin infusion)

- 5-FU 300mg/m^2 IV bolus, then 2400 mg/m^2 continuous IV infusion over 46 hours

All patients will receive supportive care as follows:

- Pegfilgrastim (Neulasta™) on day 3, 4, or 5.

- Anti-emetics including palonosetron or ondansetron, aprepitant, and dexamethasone, according to standard protocols.

- Atropine is administered per standard guidelines for cholinergic symptoms during irinotecan infusion of on day 1

- Loperamide is administered per standard guidelines for chemotherapy-induced diarrhea.

- Magnesium and calcium is administered pre- and post-oxaliplatin at the discretion of the treating physician per standard guidelines.

Patients must be counseled regarding potential sides effects and their management, including the proper use of loperamide for chemotherapy-induced diarrhea.

7.0 STUDY DRUGS/DOSE MODIFICATIONS/ TOXICITY MANAGEMENT

7.1 DOSES AND SCHEDULE OF STUDY DRUGS

Chemotherapy will consist of mFOLFIRINOX administered every two weeks, as described in detail Section 6 at the following doses.

APPROVED BY THE YALE UNIVERSITY IRB 8/7/2020

- Oxaliplatin 85 mg/m^2 IV infused over two hours, followed by
- Leucovorin 400 mg/m^2 (or levoleucovorin 200 mg/m^2) IV over two hours
- Irinotecan 135 mg/m^2 IV over 90 minutes (concurrent with leucovorin during the last 90 min of the leucovorin infusion)
- 5-FU 300mg/m^2 IV bolus, then 2400 mg/m^2 continuous IV infusion over 46 hours

7.2. STUDY DRUGS: FORMULATION, STORAGE, AVAILABILITY, PREPARATION, TOXICITY

Qualified personnel who are familiar with procedures that minimize undue exposure to themselves and to the environment should undertake the preparation, handling, and safe disposal of chemotherapeutic agents in a self-contained, protective environment.

Discard unused portions of injectable chemotherapeutic agents supplied as single-dose preparations within eight hours of vial entry to minimize the risk of bacterial contamination.

The total administered dose of chemotherapy may be rounded up or down within a range of 5% of the actual calculated dose.

7.2.1 OXALIPLATIN [ELOXATIN]

Availability

Oxaliplatin is commercially available as an aqueous solution in vials containing 50 mg and 100 mg at a concentration of 5 mg/ml. The vials do not contain any preservative and they are intended for single use.

Storage and Stability

Intact vials should be stored at room temperature. Solutions diluted in D5W are stable for 6 hours at room temperature or 24 hours under refrigeration.

APPROVED BY THE YALE UNIVERSITY IRB 8/7/2020

Preparation

The calculated dose of oxaliplatin should be diluted for infusion with 250 ml to 500 ml D5W. Oxaliplatin should not be diluted with a sodium chloride solution. Needles, syringes, catheters or IV administration sets containing aluminum should not be used with oxaliplatin. As with other platinum compounds, contact with aluminum may result in a black precipitate.

Administration

Oxaliplatin will be administered by intravenous infusion over 120 minutes in patients receiving FOlFIRINOX. Infusion time may be prolonged (up to 6 hours) in patients experiencing pharyngolaryngeal dysesthesia.

Oxaliplatin is unstable in the presence of chloride or alkaline solutions. Do NOT mix or administer oxaliplatin with saline or other chloride-containing solutions. Do NOT administer other drugs or solutions in the same infusion line. Flush IV lines/catheters with Dextrose 5% in Water both before and after oxaliplatin administration.

Toxicity

The most commonly observed oxaliplatin toxicities include neurotoxicity, GI toxicity, and myelosuppression. Three neurotoxicity syndromes have been seen:

Acute sensory neuropathy develops within hours to 2 days after oxaliplatin administration. Symptoms include paresthesia, dysesthesia, and hypothesia of the hands, feet and perioral regions. Jaw spasm, abnormal tongue sensation, dysarthria, eye pain and a sensation of chest pressure have also been noted. Acute sensory neuropathy symptoms may be exacerbated by exposure to cold temperature or cold objects. Symptoms are reversible, usually resolving within 14 days and commonly recurring with further dosing. This syndrome has been observed in about 56% of patients receiving oxaliplatin with 5-FU and leucovorin.

Acute pharyngolaryngeal dysesthesia is reported to occur in 1-2% of patients. This syndrome is characterized by a subjective sensation of difficulty breathing or swallowing without laryngospasm or bronchospasm or objective evidence of hypoxia. Avoidance of

cold drinks, food and air is suggested to order to minimize pharyngolaryngeal dysesthesia. Antianxiety agents (e.g. lorazepam) may be used to treat pharyngolaryngeal dysesthesias once oxygen saturation has been documented to be normal.

Peripheral neuropathy persisting > 14 days is characterized by paresthesias, dysesthesias, and hypothesia. Abnormalities in proprioception may also be seen. Symptoms of persistent neuropathy may improve upon discontinuation of oxaliplatin.

Various agents have been used in an attempt to minimize neurotoxicity of oxaliplatin (e.g. carbamazepine, Mg++, Ca++). Their routine use requires further confirmation of efficacy. They may be used in this study at the discretion of the treating physician.

Gastrointestinal toxicities include nausea, vomiting (oxaliplatin is considered to be moderately emetogenic) and diarrhea.

Neutropenia is reported in 73% of patients receiving oxaliplatin with 5-FU and leucovorin (44% grade 3 or 4). Grade 3 or 4 thrombocytopenia is reported to occur in 4% of patients receiving the combination.

Allergic reactions, similar to those seen with other platinum compounds, have also been observed in patients treated with oxaliplatin. Reactions range from rash to anaphylaxis

Rarely, oxaliplatin has been associated with pulmonary fibrosis, which may be fatal. Oxaliplatin should be discontinued in the presence of unexplained pulmonary symptoms (e.g. nonproductive cough, dyspnea) or pulmonary infiltrates until interstitial lung disease or pulmonary fibrosis have been ruled out.

Recent reports of oxaliplatin extravasation suggest that tissue necrosis may result and that oxaliplatin should be considered a vesicant. No standard treatment exists for oxaliplatin extravasation although heat and sodium thiosulfate have both been suggested.

Veno-occlusive disease (VOD) of the liver is a rare complication associated with oxaliplatin and 5-FU. Clinical manifestations of VOD include hepatomegaly, ascites, and jaundice. Histologically, VOD is characterized by diffuse damage in the

centrilobular zone of the liver. Sequelae of VOD include hepatomegaly, splenomegaly, portal hypertension, and esophageal varices. A recent analysis of resected liver metastases in 153 patients indicated histological findings consistent with VOD in 6/27 patients who received 5-FU alone, 4/17 patients who received 5-FU and irinotecan, 20/27 patients who received 5-FU and oxaliplatin, and 14/16 who received 5-FU, oxaliplatin and irinotecan. The remaining 66 patients had not received chemotherapy prior to resection. There were no such findings in these patients.

For more information on toxicities associated with oxaliplatin, please see the package insert.

7.2.2 5-FlUOROURACIl (5-FU: FlUOROURACIL: ADRUCIl®)

Please refer to the package insert for complete product information.

Avai/ability

5-FU is commercially available as a 50 mg/ml solution for injection in 10 ml, 20 ml, 50 ml and 100 ml vials

Preparation

Inspect for precipitate: if found, agitate or gently heat in water bath.

Bolus injections are prepared using undiluted drug.

46-48 hour infusion of 5-FU should be prepared for administration via ambulatory infusion pump according to the individual institution's standards. These solutions may be prepared in D5W or 0.9% NaCl. 5 FU should not be mixed in the same solution with most parenteral antiemetics.

Storage and Stability

Intact vials should be stored at room temperature and protected from light. Slight yellow discolor does not usually indicate decomposition. Stability in ambulatory pumps varies according to the pump, manufacturer of drug, concentration and diluent. Please refer to appropriate reference sources for additional information.

Administration

In this study, 5-FU is administered as a 300 mg/m^2 IV bolus followed by 2400 mg/m^2 by IV infusion over 46 to 48 hours.

Toxicity

Nausea, diarrhea, vomiting (mild); stomatitis (5-8 days after treatment initiation); myelosuppression; granulocytopenia (9-14 days); thrombocytopenia (7-14 days); alopecia; loss of nails; hyperpigmentation: photosensitivity; maculopapular rash; palmar-plantar erythrodysethesias: (42-82% receiving continuous infusion); CNS effects: cerebral ataxia (rare); cardiotoxicity: Ml, angina: asymptomatic S-T changes 68%; ocular effects: excessive lacrimation and less commonly, tear duct stenosis.

Drug Interactions

Leucovorin enhances the cytotoxicity of 5-FU by forming a more stable tertiary complex with thymidylate synthase. Concomitant administration of 5-FU with warfarin has been reported to result in increased INR/prolonged prothrombin time. Patients receiving both drugs should be followed with weekly INRs.

7.2.3 LEUCOVORIN CALCIUM (FOLINIC ACID; CALCIUM FOLINATE; CITROVORUM FACTOR; N 5-FORMYLTETRAHYDROFOLATE; 5-FORMYL-FH4; FOLINIC ACID).

Levoleucovorin (200 mg/m2) may be substituted for leucovorin (400 mg/m2) in this study.

Please refer to the package insert for complete product information for levoleucovorin and leucovorin.

Avai/ability

Leucovorin calcium is commercially available in: 50 mg, 100 mg, 350 mg vials for reconstitution.

Storage and Stability

Intact vials should be stored at room temperature and protected from light. Solutions reconstituted with BWI are stable for at least 7 days at room temperature.

Preparation

leucovorin may be reconstituted with Bacteriostatic Water for Injection (BWI) or with Sterile Water For Injection. Solutions should be further diluted in D5W, 0.9% NaCl or Ringers solution for infusion over two hours.

Administration

leucovorin will be administered as a 400mg/m^2 IV infusion over 2 hours after oxaliplatin administration. leucovorin may also be administered concurrently with oxaliplatin as a separate IV infusion.

Toxicity

The only adverse reactions associated with leucovorin are allergic reactions. These are extremely uncommon.

7.2.4 IRINOTECAN (CPT-11, CAMPTOSAR).

Avai/ability

lrinotecan is commercially available in a concentration of 20mg/ml in 2ml, 5ml, and 25 ml vials.

Storage and Stability

Intact vials should be stored at controlled room temperature 59 to 86 degrees Fahrenheit (15 to 30 degrees Celsius) and protected from light. Solutions diluted in D5W are reported to be stable for 48 hours under refrigeration and protected from light. lrinotecan solutions should not be frozen as the drug may precipitate.

Preparation

lrinotecan is diluted in 5% dextrose (D5W) 500 ml to a final concentration of 0.12 - 1.1 mg/ml.

Administration

In this study, irinotecan is administered by IV infusion over 90 minutes

Toxicity

Virtually all phase I and II studies of irinotecan have reported neutropenia and/or late diarrhea (diarrhea occurring more than 24 hours after the irinotecan administration as

the dose limiting toxicities (depending on the schedule)). Other commonly observed adverse events include nausea and vomiting, anorexia, abdominal cramping, alopecia, asthenia, lymphocytopenia, and anemia. Dehydration has occurred as a consequence of diarrhea, particularly when associated with severe vomiting. Patients may have an acute syndrome of lacrimation, diaphoresis, abdominal cramping, and diarrhea (early diarrhea) during or shortly after irinotecan administration; this syndrome is thought to be cholinergically mediated, and may be treated and subsequently prevented with atropine. Sporadic cases of pulmonary toxicity, manifested as shortness of breath, non-productive cough and transient infiltrates on chest x-ray have been reported. Infrequent occurrences of mucositis or colitis (sometimes with gastrointestinal bleeding) have been observed. Occasionally, abnormalities of serum creatinine, hepatic enzymes, or thrombocytopenia have been observed.

Please refer to the package insert for further information regarding irinotecan.

Concerns related to adverse effects:

• Bone marrow suppression: [U.S. Boxed Warning]: May cause severe myelosuppression. Deaths due to sepsis following severe myelosuppression have been reported. Therapy should be temporarily discontinued if neutropenic fever occurs or if the absolute neutrophil count is <1000/mm^3. The dose of irinotecan should be reduced if there is a clinically significant decrease in the total WBC (<200/mm^3), neutrophil count (<1500/mm^3), hemoglobin (<8 g/dl), or platelet count (<100,000/mm^3). Routine administration of a colony-stimulating factor is generally not necessary, but may be considered for patients experiencing significant neutropenia.

• Colitis: Colitis, complicated by ulceration, bleeding, ileus, and infection has been reported.

• Diarrhea: [U.S. Boxed Warning]: Severe diarrhea may be dose-limiting and potentially fatal; two severe (life-threatening) forms of diarrhea may occur. Early diarrhea occurs during or within 24 hours of receiving irinotecan and is characterized by cholinergic symptoms (eg, increased salivation, diaphoresis, abdominal cramping); it is usually responsive to atropine. Late diarrhea occurs more than 24 hours after treatment which may lead to dehydration, electrolyte imbalance, or sepsis; it should be promptly treated with loperamide. Patients with diarrhea should be carefully monitored and treated promptly.

APPROVED BY THE YALE UNIVERSITY IRB 8/7/2020

• Hypersensitivity reactions: Severe hypersensitivity reactions have occurred.

• Renal toxicity: Renal impairment and acute renal failure have been reported, possibly due to dehydration secondary to diarrhea.

Disease-related concerns:

• Bowel obstruction: Patients with bowel obstruction should not be treated with irinotecan until resolution of obstruction.

• Hepatic impairment: Use with caution in patients with hepatic impairment.

• Hyperbilirubinemia: Patients with even modest elevations in total serum bilirubin levels (1-2 mg/dl) have a significantly greater likelihood of experiencing first-course grade 3 or 4 neutropenia than those with bilirubin levels that were <1 mg/dl. Patients with abnormal glucuronidation of bilirubin, such as those with Gilbert's syndrome, may also be at greater risk of myelosuppression when receiving therapy with irinotecan. Use caution when treating patients with known hepatic dysfunction or hyperbilirubinemia; dosage adjustments should be considered.

7.3 DOSE MODIFICATIONS AND TOXICITY MANAGEMENT

All dose adjustments should be based on the worst preceding toxicity, graded using the National Cancer Institute (NCl)Common Terminology Criteria for Adverse Events (CTCAE) (version 4.0).

If more than one of the dose modifications apply, use the most stringent (i.e., the greatest dose reduction). Dose modifications are cumulative and permanent.

If treatment is held for any treatment-related toxicities for 3 weeks, patients should discontinue all protocol therapy.

The dose of leucovorin is not modified for toxicity, but is omitted if fluorouracil is omitted.

Dose modifications for toxicity are adapted from Conroy, T et al. FOLFIRINOX versus Gemcitabine for Metastatic Pancreatic Cancer. N Engl J Med 2011; 364:1817-1825.

APPROVED BY THE YALE UNIVERSITY IRB 8/7/2020

7.3.1 HEMATOLOGIC TOXICITY

On day 1 of treatment, hold all chemotherapy until the granulocyte count is 1500/ul and the platelet count is 75000/ul.

Doses accordinr to the blood counts at the beginning of a cycle (Day 1)

**Blood counts on Day 1**	**DELAY OF CYCLE**	**DOSE REDUCTION**		
		irinotecan	oxaliplatin	fluorouracil
**Granulocytes <1500/ul**	Hold treatment until granulocytes 1500/ul	1st occurrence: reduce to 75% of previous dose<br><br>**2nd occurrence:** maintain previous dose<br><br>3rd occurrence: treatment discontinuation	**1st** occurrence : no reduction<br><br>**2nd occurrence:** reduce to 75% of previous dose<br><br>3rd occurrence: treatment discontinuation	**1st** occurrence: delete bolus 5FU
**Platelets <75000/ul**	Hold the treatment until platelets 75000	**1st** occurrence: no reduction<br><br>2nd occurrence: reduce to 75% or original dose<br><br>**3rd 0CCUrrence:** treatment discontinuation	**1st** occurrence: reduce to 75% of previous dose<br><br>2nd occurrence: maintain previous dose<br><br>3rd **0CCUrrence:** treatment discontinuation	**1st**occurrence: reduce bolus and infusion to 75% of previous doses

Doses for neutropenic fever or nadir cytopenias

ADVERSE EVENTS	REDUCTION OF DOSE FOR SUBSEQUENT CYCLES
**Febrile neutropenia<br>or<br>Grade 4 neutropenia**	1^{s,} occurrence: reduce irinotecan to 75% of previous dose and delete the bolus 5FU dose<br><br>2nd occurrence: reduce oxaliplatin to 75% of previous dose<br><br>3rd occurrence: treatment discontinuation
**Grade 4 Thrombocytopenia**	1st occurrence: reduce oxaliplatin and 5FU (bolus and infusion) to 75% of previous dose<br><br>2nd occurrence: reduce irinotecan and 5FU (bolus and infusion) to 75% of previous dose<br><br>3rd occurrence: treatment discontinuation

7.3.2. DIARRHEA

Patients must be instructed in the use of loperamide for diarrhea, and must have a supply of this drug upon starting FOLFIRINOX. Patients should not be retreated with irinotecan until recovery from diarrhea to grade1 (without loperamide for at least 24 h) has occurred. Diarrhea is attributed to chemotherapy UNLESS a specific infectious agent is isolated.

ADVERSE EVENTS	REDUCTION OF DOSE FOR SUBSEQUENT CYCLES
**Diarrhea grade 3-4<br>or<br>Diarrhea + fever<br>and/or<br>neutropenia grade 3-4**	1^{s}•occurrence: reduce irinotecan to 75% of previous dose and delete bolus 5FU dose<br><br>2nd occurrence: reduce oxaliplatin and continuous infusion 5FU to 75% of previous doses<br><br>3rd occurrence: treatment discontinuation
**Diarrhea ::::: 48 h despite high doses loperamide**	Hold FOLFIRINOX until resolves to grade1 for at least 24 hrs.<br><br>No dose reductions after complete recovery, unless gr 3-4 diarrhea, or diarrhea + fever and/or concomitant neutropenia gr 3-4

7.7.3. MUCOSITIS OR HAND-FOOT SYNDROME

Grade 2: Hold treatment until toxicity resolves to a grade1, then resume oxaliplatin, irinotecan, and leucovorin at 100% of the previous dose and fluorouracil (bolus+ infusional 5-FU) at 75% of the previous dose for all subsequent doses. For subsequent grade.:::. 2 recurrence(s), hold treatment until toxicity resolves to a grade1, then resume oxaliplatin, irinotecan, and leucovorin at 100% of the previous dose and fluorouracil (bolus + infusional 5-FU) at 75% of the previous dose.

7.3.4. CARDIAC TOXICITY

For any cardiac arterial thrombotic event or ischemic event (angina, myocardial infarction) discontinue all protocol therapy.

7.3.5. INCREASED BILIRUBIN

In case of elevation of bilirubin, evaluation to exclude biliary obstruction or progressive disease is recommended. For bilirubin is >1.5x ULN, omit irinotecan until bilirubin is <1.5xULN.

7.3.6. NEUROTOXICITY

Toxicity Scale for the Sensory Neuropathies Associated with Oxaliplatin

	Symptoms
Grade 1	Paresthesias/dysesthesias* of short duration that resolve and do not interfere with function
Grade 2	Paresthesias/dysesthesias* interfering with function, but not with activities of daily living (AOL)
Grade 3	Paresthesias/dysesthesias* with pain or with functional impairment that also interfere with AOL.
Grade 4	Persistent paresthesias/dysesthesias* that are disabling or life threatening.
	* May be cold-induced

For grade 2 neurotoxicity persisting between treatments: Reduce oxaliplatin to75% of the previous dose for all subsequent cycles.

For grade 3 neurotoxicity resolving to grade 2 between treatments: Reduce oxaliplatin to 75% of the previous dose for all subsequent cycles.

For grade 3 neurotoxicity persisting between treatments: Discontinue Oxaliplatin. Patients should continue to receive other protocol therapy.

For grade 4 neurotoxicity: Discontinue oxaliplatin. Patients should continue to receive other protocol therapy.

7.3.7. OTHER TOXICITIES

For grade 2 toxicity attributed to treatment and not described above (except anemia and alopecia), hold treatment until toxicity resolves to grade 1 and resume treatment with the agent that is thought to be causing the toxicity at 75% of the previous dose and all other agents at full dose.

If grade 3-4 toxicity is clearly secondary to a single agent and is thought to be cumulative, the causative agent may be discontinued with the approval of the principal investigator. Similarly, a causative agent may be discontinued for grade 2 allergic reaction and must be discontinued for grade 3-4 allergic reaction.

7.3.8. EXTRAVASATION

Extravasation of oxaliplatin is reported to cause necrosis. Extravasation should be treated according to institutional guidelines.

7.3.9. DOSE MODIFICATION FOR OBESE PATIENTS

There is no clearly documented adverse impact of treatment of obese patients when dosing is performed according to actual body weight. Therefore, all dosing is to be determined solely by the patient's BSA as calculated from actual weight.

7.4 CONCOMITANT MEDICATIONS

7.4.1 Anti-emetics: Due to the emetogenic nature of this regimen, Emend will be administered with each cycle in addition to concomitant antiemetics at the discretion of the treating physician

7.4.2 Loperamide: For symptoms of diarrhea and/or abdominal cramping that occur at any time during a treatment cycle patients will be instructed to begin taking loperamide. Loperamide should be started at the earliest sign of (1) a poorly formed or loose stool or (2) the occurrence of 1 to 2 more bowel movements than usual in 1 day or (3) an increase in stool volume or liquidity. Loperamide should be taken in the following manner: 4 mg at the first onset of diarrhea, then 2 mg every 2 hours around the clock until diarrhea-free for at least 12 hours. Patients may take loperamide 4 mg every 4 hours during the night. The maximum daily dose of loperamide is 16 mg/day.

7.4.3 Antibiotics: Oral fluoroquinolone treatment may be initiated for ANC<500 or diarrhea for >24 hours despite loperamide, at the discretion of the treating physician.

7.4.4 Anticoagulants: Prophylactic or therapeutic doses of Coumadin or low-molecular weight heparin are permitted.

7.4.5 Low-dose aspirin (325 mg/d): Aspirin may be continued if the patient was on this prior to enrollment.

7.4.6 Growth Factors: Pegfilgrastim (Neulasta) will be administered with each cycle of therapy but can be omitted after the first cycle at the discretion of the treating physician for hyper-leukocytosis on day 1 of treatment.

7.4.7 Ca++/Mg++: Patients will receive supplemental calcium and magnesium infusions pre- and post-oxaliplatin at the discretion of the treating physician.

8.0 REQUIRED CLINICAL AND LABORATORY EVALUATIONS

To be completed within 14 DAYS (2 WEEKS) before the first dose of study drug:

-All blood work, History, Physical Examination, and Performance Status

To be completed within 21 DAYS (3 WEEKS) before the first dose of study drug:

-Imaging studies (CT or MRI, and PET scans)

-CT with pancreatic protocol is recommended at baseline and pre-operatively; for patients who cannot undergo CT, MRI is allowed

To be completed within 42 DAYS (6 WEEKS) before the first dose of study drug:

-Endoscopic Ultrasound (EUS)

**Tests & Observations**	**Prior to Registr-ation**	**Day 1 of each cycle* (-1/ +3 day window)**	**Day 8 of each cycle*(+/- 1 day window)**	**Pre-Operative (within 4 weeks prior to surgery)**	**Day 1 of cycle 7 (within 12 weeks of surgery)**	**Post Treatment Follow-upc** (+/- **14 day window)**
Surgical evaluation	x			x		
History	x	x			x	x
Physical Exam	x	x	xJ		x	x
Height	x					
Weight/BSA	x	x			x	x
Performance Status	x	x	xJ		x	x
Toxicity	x	x	xJ		**xx**	x
AssessmentVital SignsK	x	x	xJ			
Laboratory StudiesG:						
CBC, Diff, Platelets	x	x	x	x	x	x
Serum Chemistries+	x	x	x	x	x	x
PT/INR++	x			x		
CA 19-9, CEA	x			x	x	x
Pregnancy test#	x					
Plasma samples##	x			xF	x	xD
Archival Tissue	xi					
Staging:						
CT (or MRI)'	x			xH		xE
PET	x	xA				
Endoscopic Ultrasound (EUS)	x			xB		

* Laboratory studies must be drawn within 24 hours of administration of drugs)

\+ Serum chemistry panel will include serum creatinine, BUN, electrolytes (Na, K, Cl, Bicarb), AST/ALT, Alk Phos, Bili, and albumin

++ PT/INR should be monitored weekly for those taking coumadin or warfarin

\# For women of child-bearing potential only

\## 10-15 ml plasma for cryopreservation. This is an optional procedure. See Appendix A for additional information.

A CT prior to surgery should be performed using pancreatic protocol. For patients who are unable to undergo CT, MRI may be used

A After initial 3 cycles of neoadjuvant chemotherapy, prior to cycle 4

B Endoscopic ultrasound is optional if deemed necessary for surgical assessment

C Initiated within 4 weeks of completing all treatment and continue every 3 months for three years, then every six months for two years, then annually or until disease recurrence or death

D Plasma samples will be drawn during follow-up every 3 months for three years, then every 6 months for two years, then annually until disease progression. A sample will also be drawn at the time of disease progression.

E Initiated within 4 weeks of completing all treatment, then every 6 months for three years, and then annually for 2 years or until disease recurrence

F Plasma samples will be done post-cycle 6 and within 3-8 weeks of surgery

G Cycle 1 Day 1 laboratory studies must be done within 24 hours of the start of study drug

H CT scan will be done post-cycle 6 and within 2-8 weeks of surgery

Archival tissue from a prior biopsy may be accessed by the researchers at any time during participation on study. This is an optional procedure.

J Cycle 1 only

K Temperature, heart rate and blood pressure will be measured

9.0 CRITERIA FOR RESPONSE AND PROGRESSION

At baseline, tumor lesions will be characterized as either measurable or non-measurable.

Treating physicians and investigators will utilize RECIST version 1.1 (Eisenhauer et al. New response evaluation criteria in solid tumors: Revised RECIST guideline [version 1.1]. European Journal of Cancer 45 (2009) 228-247) in assessing response in all patients enrolled in this study, as detailed herein:

9.1 DEFINITIONS

Measurable disease:

Tumor lesions: Must be accurately measured in at least one dimension (longest diameter in the plane of measurement is to be recorded) with a minimum size of:

• 10mm by CT scan (CT scan slice thickness no greater than 5 mm).

• 10mm caliper measurement by clinical exam (lesions which cannot be accurately measured with calipers should be recorded as non-measurable).

• 20mm by chest X-ray.

Malignant lymph nodes: To be considered pathologically enlarged and measurable, a lymph node must be 15mm in short axis when assessed by CT scan (CT scan slice

thickness recommended to be no greater than 5 mm). At baseline and in follow-up, only the short axis will be measured and followed.

Non-measurable disease:
All other lesions, including small lesions (longest diameter <10mm or pathological lymph nodes with .:::_10 to <15mm short axis) as well as truly non-measurable lesions. Lesions considered truly non-measurable include: leptomeningeal disease, ascites, pleural or pericardia! effusion, inflammatory breast disease, lymphangitic involvement of skin or lung, abdominal masses/abdominal organomegaly identified by physical exam that is not measurable by reproducible imaging techniques.

9.2 METHODS OF MEASUREMENT

The same method of assessment and the same technique should be used to characterize each identified and reported lesion at baseline and during follow-up. Imaging based evaluation should always be done rather than clinical examination unless the lesion(s) being followed cannot be imaged but are assessable by clinical exam.

Computed tomography (CT)/MRI - CT and MRI might be the best currently available and reproducible methods to measure target lesions selected for response assessment. Conventional CT and MRI should be performed with cuts of 5 mm or less in slice thickness contiguously. Spiral CT should be performed using a 5-mm contiguous reconstruction algorithm.

9.3 BASELINE DOCUMENTATION OF TARGET AND NON-TARGET LESIONS

Target lesions:
When more than one measurable lesion is present at baseline all lesions up to a maximum of five lesions total (and a maximum of two lesions per organ) representative of all involved organs should be identified as target lesions and will be recorded and measured at baseline (this means in instances where patients have only one or two organ sites involved a maximum of two and four lesions respectively will be recorded).

Lymph nodes: These merit special mention since they are normal anatomical structures which may be visible by imaging even if not involved by tumor. Pathological nodes

which are defined as measurable and may be identified as target lesions must meet the criterion of a short axis of 15mm by CT scan. Only the short axis of these nodes will contribute to the baseline sum. The short axis of the node is the diameter normally used by radiologists to judge if a node is involved by solid tumor. Nodal size is normally reported as two dimensions in the plane in which the image is obtained (for CT scan this is almost always the axial plane; for MRI the plane of acquisition may be axial, saggital or coronal). The smaller of these measures is the short axis. All other pathological nodes (those with short axis 10mm but <15 mm) should be considered non-target lesions. Nodes that have a short axis <10mm are considered non-pathological and should not be recorded or followed.

A sum of the diameters (longest for non-nodal lesions, short axis for nodal lesions) for all target lesions will be calculated and reported as the baseline sum diameters. If lymph nodes are to be included in the sum, then as noted above, only the short axis is added into the sum. The baseline sum diameters will be used as reference to further characterize any objective tumor regression in the measurable dimension of the disease.

Non-target lesions:
All non-measurable lesions (or sites of disease) plus any measurable lesions over and above the 5 listed as *target lesions.* Measurements are not required but these lesions should be noted at baseline and should be followed as "present" or "absent."

9.4 EVALUATION OF BEST OVERALL RESPONSE

In general, the patient's best response assignment will depend on the achievement of both measurement and confirmation criteria as outlined below and summarized in Table 1:

Complete Response (CR): Disappearance of all target lesions.
Any pathological lymph nodes (whether target or non-target) must have reduction in short axis to <10 mm.

Partial Response (PR): At least a 30% decrease in the sum of diameters of target lesions, taking as reference the baseline sum diameters.

APPROVED BY THE YALE UNIVERSITY IRB 8/7/2020

Progressive Disease (PD): At least a 20% increase in the sum of diameters of target lesions, taking as reference the smallest sum on study (this includes the baseline sum if that is the smallest on study). In addition to the relative increase of 20%, the sum must also demonstrate an absolute increase of at least 5 mm. (Note: the appearance of one or more new lesions is also considered progression).

Stable Disease (SD): Neither sufficient shrinkage to qualify for PR nor sufficient increase to qualify for PD, taking as reference the smallest sum of diameters while on study.

Table 1: Response Assessment in Patients with Measurable Disease

Target Lesions	Non-Target Lesions	New Lesions	Overall Response	Best Response for this Category also requires
CR	CR	No	CR	≥4 weeks Confirmation
CR	Non-CR/Non-PD	No	PR	≥4 weeks Confirmation
PR	Non-PD	No	PR	
SD	Non-PD	No	SD	Documented at least once > 6 weeks from baseline
PD	Any	Yes or No	PD	No prior SD, PR or CR
Any	PD	Yes or No	PD	
Any	Any	Yes	PD	

Every effort should be made to document the objective progression even after discontinuation of treatment

Response duration will be measured from the time measurement criteria for CR/PR (whichever is first recorded) are first met until the first date that recurrent or PD is objectively documented.

Stable disease duration will be measured from the time of start of therapy until the criteria for progression are met, taking as reference the smallest measurements recorded since the treatment started.

9.5 EVALUATION OF RESPONSE FOR NON-MEASURABLE DISEASE

When the patient has only non-measurable disease, the same general concepts apply here as noted above. However, in this instance there is no measurable disease assessment to factor into the interpretation of an increase in non-measurable disease burden. Because worsening in non-target disease cannot be easily quantified (by definition: if all lesions are truly non-measurable) a useful test that can be applied when assessing patients for unequivocal progression is to consider if the increase in overall disease burden based on the change in non-measurable disease is comparable in magnitude to the increase that would be required to declare PD for measurable disease: i.e. an increase in tumor burden representing an additional 73% increase in 'volume' (which is equivalent to a 20% increase diameter in a measurable lesion). Examples include an increase in a pleural effusion from 'trace' to 'large', an increase in lymphangitic disease from localized to widespread, or may be described in protocols as 'sufficient to require a change in therapy'. If 'unequivocal progression' is seen, the patient should be considered to have had overall PD at that point. While it would be ideal to have objective criteria to apply to non-measurable disease, the very nature of that disease makes it impossible to do so, therefore the increase must be substantial.

It is assumed that at each protocol specified time point, a response assessment occurs. When patients have non-measurable (therefore non-target) disease only, Table 2 is to be used.

Table 2 – Time point response: patients with non-target disease only.

Non-target lesions	New lesions	Overall response
CR	No	CR
Non-CR/non-PD	No	Non-CR/non-PD[a]
Not all evaluated	No	NE
Unequivocal PD	Yes or No	PD
Any	Yes	PD

CR = complete response, PD = progressive disease, and NE = inevaluable.

a 'Non-CR/non-PD' is preferred over 'stable disease' for non-target disease since SD is increasingly used as endpoint for assessment of efficacy in some trials so to assign this category when no lesions can be measured is not advised.

10.0 SUBJECT DISCONTINUATION

Treatment will continue for 6 cycles (12 weeks) pre-operatively and 6 cycles (12 weeks) post-operatively, or until disease progression, or until unacceptable toxicity as specified in the protocol (section 7.3). Patients who are deemed unresectable at the time of surgery will be discontinued from study treatment.

Patients who complete the study treatment phase without disease progression will enter a post-study surveillance phase. They will undergo history, physical, and lab assessments at three month intervals and cross-sectional imaging at 6 months intervals for three years, and then at six month intervals for two additional years. After five years, patients will be followed annually until death; surveillance imaging and lab assessments are optional after five years.

In addition, subjects who meet the following criteria should be discontinued from study treatment:

- Any grade 3 or 4 toxicity that does not resolve within 3 weeks of holding treatment.
- Unwillingness or inability of subject to comply with study requirements
- Determination by the investigator that it is no longer safe for the subject to continue therapy, or that the constraints of this protocol are detrimental to the patient's health
- The patient no longer wishes to continue protocol therapy

All subjects will be followed for survival.

11.0 STUDY DISCONTINUATION

The Principal Investigator has the right to terminate this study at any time if the incidence or severity of adverse events in this or other studies indicates a potential health hazard to subjects.

12.0 STATISTICAL METHODS

The primary objective of this phase II study is to determine the proportion of patients who are alive and free from progression of disease at 12 months from the first cycle of neoadjuvant mFOLFIRINOX. Thus, the primary endpoint of this study is 12-month PFS rate in patients with resectable non-metastatic pancreatic cancer after receiving modified FOLFIRINOX in the neoadjuvant and adjuvant settings. With the current standard of care (adjuvant gemcitabine) patients would be expected to have a progression-free survival rate at 12 months of 54% (the corresponding median PFS is 13.4 months) (2). Alternatively, the regimen (peri-operative mFOLFIRINOX) will be considered worthy of further study if its true progression-free survival rate at 12 months is 66% or better (the corresponding median PFS is 19.7 months).

We will enroll 46 patients in this trial. Using a one-sided 0.10-alpha level exact test, we will have approximately 82% power to detect a survival proportion at 12 months of 0.66 assuming that the PFS proportion at 12 months under the null hypothesis is 0.50. Therefore, this treatment can be considered for further investigation if at least 28 of the 46 patients are alive at 12 months without progression.

Upon completion of the study we will summarize PFS and overall survival using Kaplan-Meier curves. Rates of toxicity will be reported along with exact binomial confidence intervals. We will use proportional hazards regression to determine if overall survival and PFS are related to the occurrence of toxicity or demographic variables.

Any additional correlative studies (PET scan) will include proportional hazards regressions to determine if there are demographic or clinical parameters that can be used to identify patients with longer or shorter anticipated PFS or OS. This trial is not powered for these correlative secondary outcomes.

We anticipate accrual will be completed in five years, based on the number of pancreatic resections for pancreatic adenocarcinoma that are performed annual at Yale New Haven Hospital annually (>40 per year).

13.0 ADVERSE EVENT ANALYSIS, DEFINITION, AND REPORTING

13.1 SAFETY ANALYSIS

Safety will be analyzed for patients treated in this study.

At each visit, a brief focused history will be obtained and any indication of treatment related toxicity will be evaluated by appropriate examination and/or laboratory/radiographic studies.

Safety analyses will include summaries of adverse event rates and changes in laboratory results, as well as number of CTCAE toxicity grades for both laboratory and non-laboratory data.

The evaluation period should extend from date of first treatment until at least 30 days (or longer if so specified) from the last dose or until resolution from all acute toxicities associated with the drug administration.

13.2 DEFINITION OF ADVERSE EVENT TERMS

Adverse Event - Any unfavorable and unintended sign (including an abnormal laboratory finding), symptom, or disease temporally associated with the use of a medical treatment or procedure regardless of whether it is considered related to the medical treatment or procedure (attribution of unrelated, unlikely, possible, probable, or definite). [NIH Guidelines, January 2001]

Serious Adverse Event (SAE) - Any adverse drug experience occurring at any dose that results in any of the following outcomes:

- death,
- a life-threatening adverse drug experience,
- in patient hospitalization or prolongation of existing hospitalization,
- any persistent or significant disability/incapacity,
- or a congenital anomaly/birth defect.

Important medical events that may not result in death, be life-threatening, or require hospitalization may be considered a serious adverse drug experience when, based upon appropriate medical judgment, they may jeopardize the patient or subject and may require medical or surgical intervention to prevent one of the outcomes listed in this definition.

"Serious" Versus "Severe" Adverse Events - There is a distinction between serious and severe AEs. Assessment of seriousness will be made solely by the serious criteria listed above. Severity of AEs will be graded according to the NCI Common Terminology Criteria for Adverse Events v3.0. Therefore, serious events will not be automatically considered severe. For example, a stroke that results in only a limited degree of disability may be considered a mild (not severe) stroke, but it would still meet serious criteria and thus, be captured as an SAE. Similarly, severe events may not always be serious. An example would be an episode of severe, transient nausea which persists for several hours. This would be classified as a "severe" episode of nausea, but if it did not require treatment, intervention, or somehow meet other serious criteria, it would not be considered an SAE.

Life-threatening Adverse Drug Experience - Any adverse drug experience that places the patient or subject, in the view of the investigator, at immediate risk of death from the

APPROVED BY THE YALE UNIVERSITY IRB 8/7/2020

reaction as it occurred, i.e. it does not include a reaction that, had it occurred in a more severe form, might have caused death.

Unexpected Adverse Drug Experience - Any adverse drug experience, the specificity or severity of which is not consistent with the current investigator brochure; or, if an investigator brochure is not required or available, the specificity or severity of which is not consistent with the risk information described in the general investigational plan. "Unexpected" as used in this definition, refers to an adverse drug experience that has not been previously observed (e.g., included in the investigator brochure) rather than from the perspective of such experience not being anticipated from the pharmacological properties of the pharmaceutical product.

13.3 TOXICITY GRADING

Toxicities will be graded according to the current version of the NCI Common Terminology Criteria for Adverse Events (CTCAE) Version 4.0. The full text of the NCI CTCAE is available online at: http://evs.nci.nih.gov/ftp1/CTCAE/CTCAE_4.03_2010-06-14_QuickReference_5x7.pdf

If a certain event or symptom is not described in the CTCAE grades, use the following grading scale:

- Mild: awareness of event but easily tolerated
- Moderate: discomfort enough to cause some interference with usual activity
- Severe: inability to carry out usual activity
- Very Severe: debilitating, significantly incapacitates patient despite symptomatic therapy

13.4 TOXICITY ATTRIBUTION

Assessment of attribution is made by consideration of all clinically relevant data prior to, during, and after occurrence of the event, including diagnostic tests to assess the cause of the event. Clinically relevant data include, but are not limited to; underlying disease, past and present medical history (all concurrent non-malignant disease), concurrent medications, and timing between event and drug administration. The mechanism of action and prior toxicology of the study drug should be considered.

APPROVED BY THE YALE UNIVERSITY IRB 8/7/2020

An adverse event is associated with the use of the drug when there is a reasonable possibility that the experience may have been caused by the drug.

Attribution Standards per NCI - CTEP:

Unrelated: The Adverse Event is clearly not related to the investigational agent(s) Unlikely: The Adverse Event is doubtfully related to the investigational agent(s)
Possible: The Adverse Event may be related to the investigational agent(s)
Probable: The Adverse Event is likely related to the investigational agent(s)
Definite: The Adverse Event is clearly related to the investigational agent(s)

14.0 YALE PRINCIPAL INVESTIGATOR SAE REPORTING REQUIREMENTS

14.1 EXPEDITED REPORTING OF UNEXPECTED SAES

AEs classified as "serious" and "unexpected" that are possibly, probably, or definitely attributed to drug administration, or SAEs whose frequency exceeds expectations, require expeditious handling and reporting.

The PI will promptly investigate all safety information related to an adverse experience. If the results of the PI's investigation show an adverse drug experience not initially determined to be reportable (based on whether the event is serious, unexpected, and associated with drug administration) is so reportable, the PI will report such experience. Follow-up information to a safety report shall be submitted as soon as the relevant information is available.

<u>Reporting to the Yale Human Investigation Committee</u>

All SAEs, whether originating at Yale or a collaborating center, meeting the criteria for expedited reporting will be reported to the Yale University Human Investigation Committee (HIC) using HIC <u>Form 710 FR 4</u> as per IRB Policy 710.

The Yale University Human Investigation Committee expedited reporting criteria are:

a. Serious AND unanticipated AND possibly, probably or definitely related events;

and

b. Anticipated Adverse Events occurring with a greater frequency than expected.

The HIC does not require reporting of any other Adverse Event type. A copy of the Yale IRBPolicy 710 Reporting Unanticipated Problems Involving Risks to Subjects or Others, including Adverse Events is available at: http://www.yale.edu/hrpp/policies/documents/NewlRBPolicy710_UPIRSOSAE_4.3.2014_withflowchart_vF.PDF

14.2 DURATION OF REPORTING OF SAES

From the date of first treatment until 30 days (unless otherwise specified) subsequent to last treatment or withdrawal of subject, new onset adverse events will be captured. Follow-up and reporting of these events will follow the same procedure as for AEs observed during the study period. In addition, any unexpected Serious Adverse Event that occurs more than 30 days after drug administration but is possibly, probably or definitely attributed to drug administration will be recorded and reported.

15.0 YALE SAFETY REPORTING AND MONITORING (DSMP)

The principal investigator at the Yale Cancer Center will monitor the clinical trial for safety. The principal investigator will assess all expedited adverse events and will periodically review all adverse events observed on the trial. Yale Cancer Center standard operating procedures (SOPs) for assessment and reporting of adverse events are followed which are in compliance with FDA 21 Code of Federal Regulations Part 312.32 and 312.33.

The clinical trial data consisting of all required observations, AEs, and laboratory data will be entered into a computerized database (OnCore) in a timely manner. The accuracy and completeness of the database, timely submission of SAEs and compliance with the protocol, is assured by periodic auditing conducted by the Yale Cancer Center Office of Protocol Review and Monitoring, which reports to the Yale Data and Safety Monitoring Committee (DSMC). Safety data will be submitted to DSMC at

least once yearly or more often as required by the DSMC. On a regular interval basis, status reports of all laboratory parameters, AEs and SAEs are reviewed by the PI to view composite data across subjects. Regular meetings are held to discuss ongoing patient treatment and adverse events.

The Yale DSMC will perform an interim safety analysis after enrollment of 20 patients to determine whether there are any safety issues and to assess whether it is safe to continue enrollment. Enrollment will be suspended until approval is granted from the DSMC to proceed completion of enrollment.

Possible actions taken by the PI or the Yale DSMC if a new unexpected toxicity is identified from the above safety review, or if the periodic review of all adverse events and laboratory data indicates a pattern of incidence or severity of toxicity that raises a safety concern, can be to:

- Revise consent form
- Amend the protocol
- Suspend the protocol

All AEs found to be expected or non-serious, will be included in the Annual Report.

16.0 RETENTION OF RECORDS

U.S. FDA regulations (21 CFR 312.62[c]) require that records and documents pertaining to the conduct of this study and the distribution of investigational drug, including CRFs, consent forms, laboratory test results, and medication inventory records, must be retained by the principal investigator for 2 years after the investigation is discontinued.

17.0 RESEARCH ETHICS AND HUMAN SUBJECT PROTECTION

17.1 SUBJECT SELECTION

This protocol will seek to accrue patients 18 years of age or older as described in the study inclusion criteria.

17.2 INFORMED CONSENT PROCESS

The informed consent dialogue with the research subjects will include a discussion of the natural history of their condition and alternative therapies that a

person might consider (e.g. supportive care only, administration of conventional chemotherapy agents, or participation in a research study).

17.3 CONFIDENTIALITY

Data collected on each patient will be maintained in a research folder kept in a locked room. Data entered into a computerized database will be kept on a computer that is password protected and kept in a locked room. In all publications and presentations resulting from this research project, the research subject's anonymity will be protected to the maximum extent possible. Authorized Medical Department personnel and personnel from the cancer center and IRB may have access to their research file in order to verify that the research subject's rights have been safeguarded. In addition, their name will be given to the Clinical Studies Unit, a contracting office who will register the research subject onto this study and verify their eligibility.

17.4 PATIENT'S RIGHTS

If the research subject suffers any physical injury as a result of their participation in this study, immediate medical treatment is available at the treating institution. Although no compensation is available, any injury as a result of his participation will be evaluated and treated in keeping with the benefits or care to which he is entitled under applicable regulations.

If the research subject has any questions regarding this research project, he may contact Dr. Jill Lacy or site investigator or coordinator or IRB. If he has any questions regarding his rights as an individual while participating in a research project at the Yale Cancer Center (YCC), he can contact one of the Research Administrators, Clinical Investigation Department, who will answer the research subject's questions or refer him to a member of the committee for the Protection of Human Subjects for further information. If the patient believes that he has been injured as a result of this project, he may call the legal office of YCC.

Participation in this research project is voluntary. A patient's refusal to participate will involve no penalty or loss of benefits to which he is entitled under applicable regulations. If the chooses to participate, he is free to ask questions or to withdraw from the project at any time. If he should decide to withdraw from

the research project, he will notify the PI either directly or indirectly through the research nurse, to make sure the reasons for withdrawal are recorded and to ensure that everything is in order. A patient's withdrawal will involve no loss of benefits to which he is entitled.

The investigators may terminate the research subject's participation in this project for the following reasons: growth of his cancer, intolerance of treatment, inability to comply with the protocol guidelines for treatment and follow-up, or if the patient chooses to stop protocol therapy, or at the specific request of MEI. Any new significant finding developed during the course of the research which may affect his willingness to participate further will be explained to the research subject.

18.0 REFERENCES

1. Jemal A, Siegel R, Xu J, Ward E. Cancer statistics, 2010. CA Cancer J Clin 2010;60:277-300.

2. Oettle H et al. Adjuvant chemotherapy with gemcitabine vs observation in patients undergoing curative-intent resection of pancreatic cancer. JAMA 2007; 297(3)267-277.

3. Neoptolemos JP, Stacken DD, Friess H, et al. A randomized trial of chemoradiotherapy and chemotherapy after resection of pancreatic cancer. N Engl J Med. 2004 Mar 18;350(12):1200-10.

4. Neoptolemos, JP et al. Adjuvant chemotherapy with fluorouracil plus folinic acid vs gemcitabine following pancreatic cancer resection: a randomized controlled trial. JAMA. 2010:304(10) 1073-81.

5. Takai S, Satoi S, Toyokawa H, Yanagimoto H, Sugimoto N, Tsuji K, et al. Clinicopathologic evaluation after resection for ductal adenocarcinoma of the pancreas: a retrospective, single-institution experience. Pancreas. 2003;26:243-9.

6. Neoptolemos JP, Stacken DD, Dunn JA, Almond J, Beger HG, Pederzoli P, et al. Influence of resection margins on survival for patients with pancreatic cancer treated by

adjuvant chemoradiation and/or chemotherapy in the ESPAC-1 randomized controlled trial. Ann Surg. 2001;234:758-68.

7. Sohn TA, Yeo CJ, Cameron JL, Koniaris L, Kaushal S, Abrams RA, et al. Resected adenocarcinoma of the pancreas-616 patients: results, outcomes, and prognostic indicators. J Gastrointest Surg. 2000;4:567-79.

8. Neuhaus P, Riess H, Post S, et al. CONKO-001: Final results of the randomized, prospective, multicenter phase Ill trial of adjuvant chemotherapy with gemcitabine vs. observation in patients with resected pancreatic cancer (abstract). J Clin Oneal 2008; 26:1009s

9. Conroy T, Desseigne F, Ychou **M,** et al. FOLFIRINOX versus gemcitabine for metastatic pancreatic cancer. N Engl J Med 2011;364:1817-25.

10. Gunturu KS, Thumar JR, Hochster HS, Stein S, Yao X, Cong X, Hahn C, Kaley K, Lacy J. Single-institution experience with FOLFIRINOX in advanced pancreatic cancer (PC). J Clin Oneal 30, 2012 (suppl 4; abstr 330)

11. Gunturu KS, Yao X, Cong X, Thumar JR, Hochster HS, Stein SM, Lacy J. FOLFIRINOX for Locally Advanced and Metastatic Pancreatic Cancer: Single Institution Retrospective Review of Efficacy and Toxicity Medical Oncology (in press)

12. Spitz FR, Abbruzzese JL, Lee JE, Pisters PW, Lowy AM, Fenoglio CJ, Cleary KR, Janjan NA, Goswitz MS, Rich TA, Evans DB. Preoperative and postoperative chemoradiation strategies in patients treated with pancreaticoduodenectomy for adenocarcinoma of the pancreas. J Clin Oncol. 1997 Mar;15(3):928-37.

13. Varadhachary GR, Tamm EP, Abbruzzese JL, et al. Borderline resectable pancreatic cancer: definitions, management, and role of preoperative therapy. Ann Surg Oneal 2006;8:1035-1046

14. Varadhachary GR et al. Preoperative gemcitabine and cisplatin followed by gemcitabine-based chemoradiation for resectable adenocarcinoma of the pancreatic head. J Clin Oncol 2008; 26(21): 3487-3495.

15. Evans DB et al. Preoperative gemcitabine-based chemoratiation for patients with resectable adenocarcinoma of the pancreatic head. J Clin Oncol 2008; 26(21) 3496- 3502.

16. Palmer DH, Stacken DD, Hewitt H, Markham CE, Hassan AB, Johnson PJ, Buckels JA, Bramhall SR. A randomized phase 2 trial of neoadjuvant chemotherapy in resectable pancreatic cancer: gemcitabine alone versus gemcitabine combined with cisplatin. Ann Surg Oncol. 2007 Jul;14(7):2088-96. Epub 2007 Apr 24.

17. Heinrich S, Pestalozzi BC, Schafer M, Weber A, Bauerfeind P, Knuth A, Clavien PA. Prospective phase II trial of neoadjuvant chemotherapy with gemcitabine and cisplatin for resectable adenocarcinoma of the pancreatic head. J Clin Oncol. 2008 May 20;26(15):2526-31.

18. Varadhachary GR. Preoperative therapies for resectable and borderline pancreatic cancer. J Gastrointest Oncol 2011; 2:136-142.

19. Le Scodan R, Mornex F, Girard N, Mercier C, Valette PJ, Ychou M, Bibeau F, Roy P, Scoazec JY, Partensky C. Preoperative chemoradiation in potentially resectable pancreatic adenocarcinoma: feasibility, treatment effect evaluation and prognostic factors, analysis of the SFRO-FFCD 9704 trial and literature review. Ann Oncol. 2009 Aug;20(8):1387-96. Epub 2009 Jun 5.

20. Talamonti MS, Small W Jr, Mulcahy MF, Wayne JD, Attaluri V, Colletti LM, Zalupski MM, Hoffman JP, Freedman GM, Kinsella TJ, Philip PA, McGinn CJ. A multi-institutional phase II trial of preoperative full-dose gemcitabine and concurrent radiation for patients with potentially resectable pancreatic carcinoma. Ann Surg Oncol. 2006 Feb;13(2):150-8. Epub 2006 Jan 19.

APPROVED BY THE YALE UNIVERSITY IRB 8/7/2020

APPENDIX A: STANDARD PROCEDURE FOR COLLECTION AND STORAGE OF PLASMA AND BUFFY COAT SPECIMENS

1. PURPOSE

Whole blood is collected from patients to bank plasma in 200ul and 1ml aliquots along with white blood cells (buffy coat). Collection will occur at baseline (3 tubes or 30 mls) and then 1-2 tubes (or 10-20 mls) each at the specified Pre-Operative visit, the specified Post-Operative, and at each specified Follow Up visit.

2. MATERIALS

- Becton-Dickinson Vacutainer *K2* EDTA (10 ml) Ref 3666432ml and 0.5 ml micro tubes (Sarstedt Ref 72.730.005 and 72.694.005) P200 and Pl000 micro pipettes and aerosol barrier tips low speed tabletop centrifuge

3. PROTOCOL

1.) Collect 3 tubes of blood at baseline and 1 or 2 tubes at the subsequent visits from consented patients. Once blood tubes are back in the lab, allow them to sit until separation of the two layers begins to occur.
   a. Store at room temperature until preparing the samples.
   b. Process the samples within 4 hours of being drawn.
   c. Enter information regarding time/day etc. of obtaining into a plasma tracking sheet form.

2.) Within 4 hours of collection spin the purple top blood collection tubes in the centrifuge (1000 - 1200g) for 10 minutes.

3.) While the blood is spinning, label cryotubes for 10 of the 1ml tubes of plasma, 3 of the 2ml tubes for buffy coats and 3 of the 10 of the 2ml tubes for plasma, depending on the volume of blood.
   Include the following information:
   a. Study HIC# 2255
   b. Patient ID PT xxxxxx
   c. Plasma or buffy coat
   d. Visit name and date of collection
   e. How much is in the tube.g. 200µ1 or 1.8 ml
   f. The number of the tube 001, 002 etc.

APPROVED BY THE YALE UNIVERSITY IRB 8/7/2020

4.) Gently remove EDTA tubes from the centrifuge so as not to disturb the huffy coat.

5.) Pipette 1000µ1 into each 2 ml tube for a final volume of 1ml (2-3 aliquots).

6.) Pipette 200µ1 of plasma into each of the **1 ml** tubes.

7.) Once all possible aliquots are removed from blood collection tubes, take the P200 and pipette the remaining plasma without disturbing the white huffy coat layer.

8.) Using the P200 carefully remove the huffy coat (white layer) from each blood collection tube without removing too many red blood cells below.

9.) Once the huffy coat is removed, place the cap back on the EDTA tube and place in sharps container.

Place the plasma and huffy coat aliquots in the appropriate box in the -80°C freezer.
