# Supplementary material for: Perioperative Modified FOLFIRINOX for Resectable Pancreatic Cancer: A Nonrandomized Controlled Trial
Source: JAMA Oncol. 2024 Jun 20;10(8):1027–35. doi: 10.1001/jamaoncol.2024.1575 (PMC11190830; doi:10.1001/jamaoncol.2024.1575)
Supplement: Supplement 2. — eMethods. eFigure 1. Waterfall Plot eTable 1. Surgical Outcomes eFigure 2. Supplementary Keratin 17 Signatures eFigure 3. Mutational Signatures for All Enrolled Patients eTable 2. Frequency of Treatment Emergent Adverse Events and Laboratory Abnormalities With ≥10% Incidence eFigure 4. Mutational Signatures for Baseline ctDNA-Negative and ctDNA-Positive Cohorts eFigure 5. Mutational Signatures for Exceptional, Intermediate, and Poor Responders [file jamaoncol-e241575-s002.pdf]

Supplemental Online Content

Cecchini M, Salem RR, Robert M, et al. Perioperative modified FOLFIRINOX for resectable pancreatic cancer: a nonrandomized controlled trial. *JAMA Oncol*. Published online June 20, 2024. doi:10.1001/jamaoncol.2024.1575

eMethods.
eFigure 1. Waterfall Plot
eTable 1. Surgical Outcomes
eFigure 2. Supplementary Keratin 17 Signatures
eFigure 3. Mutational Signatures for All Enrolled Patients
eTable 2. Frequency of Treatment Emergent Adverse Events and Laboratory Abnormalities With ≥10% Incidence
eFigure 4. Mutational Signatures for Baseline ctDNA-Negative and ctDNA-Positive Cohorts
eFigure 5. Mutational Signatures for Exceptional, Intermediate, and Poor Responders

This supplementary material has been provided by the authors to give readers additional information about their work.

© 2024 Cecchini M et al. *JAMA Oncology*.

eMethods.

Study Assessments:

Participants underwent a baseline pancreatic protocol CT (or MRI), PET/CT, and endoscopic ultrasound. A staging laparoscopy was not required. A PET/CT was repeated after 3 cycles and repeat pancreatic protocol CT (or MRI) was repeated after 6 cycles to confirm sustained operative candidacy.

Plasma Collection:

Approximately 30 ml of whole blood was collected in EDTA tubes and processed to plasma for storage in a -80°C freezer at the following time points: 1) baseline: prior to treatment 2) preoperative: after 6 cycles of mFOLFIRINOX 3) postoperative: 3-8 weeks after surgery before starting adjuvant mFOLFIRINOX 4) end of treatment: after 6 cycles of adjuvant mFOLFIRINOX and 5) at radiographic progression.

Statistical Software:

Statistical analyses for the clinical endpoints were performed using R version 4.1.2. Sample size estimation was performed using PASS version 21.0.5.

© 2024 Cecchini M et al. *JAMA Oncology*.

eFigure 1. Waterfall Plot

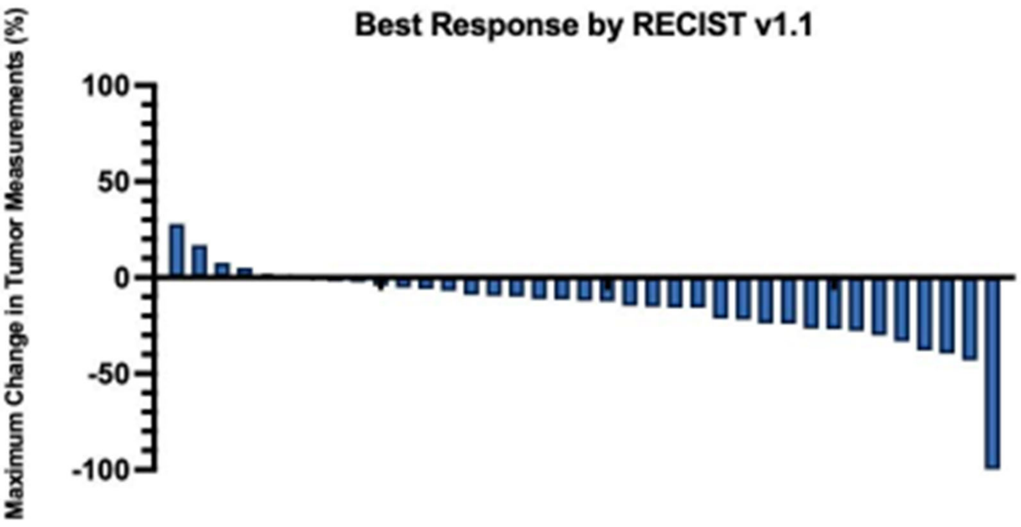

Best response by RECIST version 1.1 in patients with measurable disease.

© 2024 Cecchini M et al. *JAMA Oncology*.

eTable 1. Surgical Outcomes

Characteristic	Surgery Resection (N=27)
Pathologic Stage	
IA	4 (15)
IB	2 (7)
IIA	4 (15)
IIB	17 (63)
Resection margins	
R0	25 (93)
R1	2 (7)
Lymphovascular Invasion	
Present	15 (56)
Absent	12 (44)
Type of Operation	
Standard Pancreaticoduodenectomy	13 (48)
Pylorus Preserving PD	7 (26)
Distal Pancreatectomy	7 (26)
Total Pancreatectomy	0
Vascular Resection	
Yes	1
No	26
Resection	
Total nodes (Median, range)	27 (6-41)
Positive nodes (Median, range)	1 (0-11)
0 nodes positive	10
1-3 nodes positive	11
>3 nodes positive	6
Pathologic response	
0	0 (0)
1	3 (11)
2	6 (22)
3	16 (59)
Unable to assess	2 (8)

At least one dose reduction of mFOLFIRINOX was required in 7 of 22 patients (32%) in the post operative period and two patients discontinued adjuvant mFOLFIRINOX for treatment related adverse events.

© 2024 Cecchini M et al. *JAMA Oncology*.

eFigure 2. Supplementary Keratin 17 Signatures

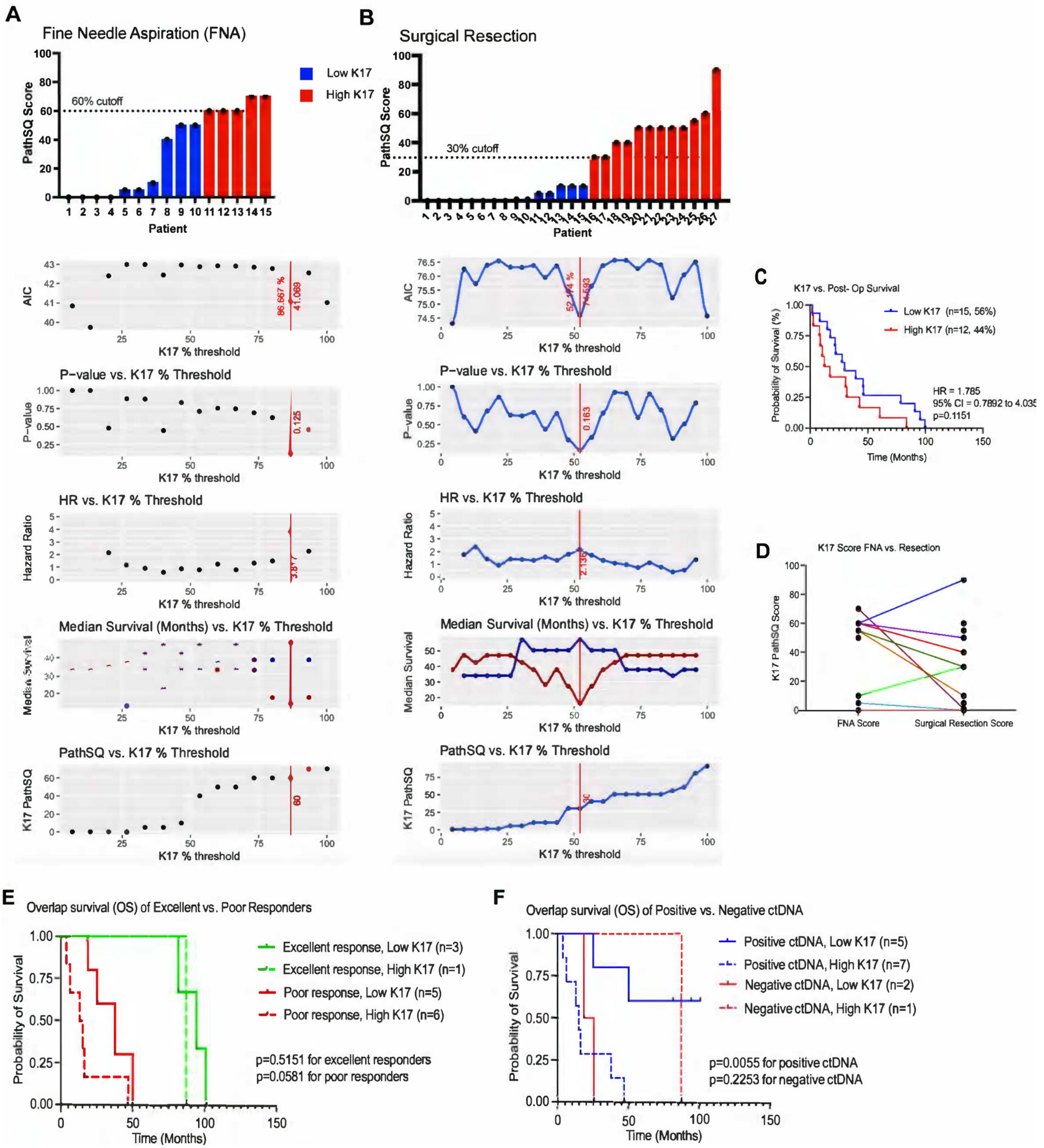

A-B. PathSQ score distribution and AIC analysis needle aspirates/biopsies (**A**) and surgical specimens (**B**). Post-operation survival (**C**). D. K17 PathSQ scores in matched samples from needle aspirates/biopsies and surgical spcimens. **E.** Interaction of therapy response status and K17 status in overall survival. Poor response was defined as median OS <25 months and excellent response as median OS ≥ 25 months. **F.** Interaction of ctDNA and K17 status in overall survival.

© 2024 Cecchini M et al. *JAMA Oncology*.

© 2024 Cecchini M et al. *JAMA Oncology*.

eFigure 3. Mutational Signatures for All Enrolled Patients

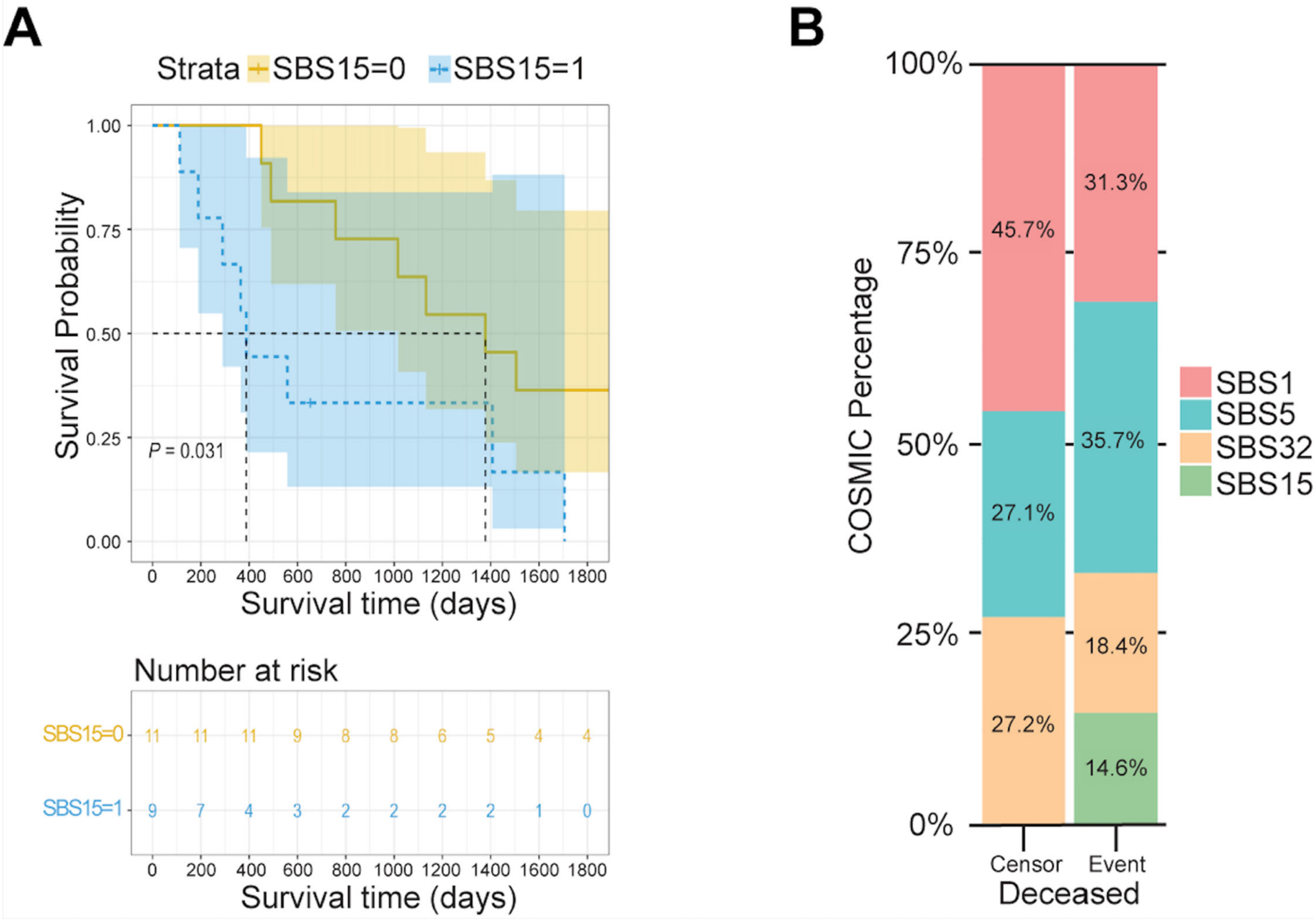

A. Survival by SBS15 mutational signature. **B.** Cosmic percentage of signature.

© 2024 Cecchini M et al. *JAMA Oncology*.

eTable 2. Frequency of Treatment Emergent Adverse Events and Laboratory Abnormalities With ≥10% Incidence

Event	Any Grade	Grade ≥ 3
Any event – no. (%)	46 (100)	35 (76)
Any serious event – no. (%)[1]	13 (28)	13 (28)
Most common events – no. (%)		
Fatigue	36 (78)	3 (7)
Diarrhea	30 (65)	4 (9)
Nausea	27 (59)	2 (4)
Paresthesia	27 (59)	0
Anorexia	21 (46)	0
Neuropathy	20 (43)	3 (7)
Abdominal pain	19 (41)	2 (4)
Constipation	16 (35)	0
Mucositis	15 (33)	0
Weight loss	15 (33)	0
Vomiting	15 (33)	0
Flatulence	14 (30)	0
Dysguesia	14 (30)	0
Edema	9 (20)	0
Insomnia	8 (17)	0
Alopecia	8 (17)	0
Dizziness	7 (15)	0
Hypertension	7 (15)	3 (7)
Hyponatremia	6 (13)	1
Thromboembolism	6 (13)	2 (4)
Back pain	6 (13)	0
Headache	5 (11)	0
Anxiety	5 (11)	0
Hiccups	5 (11)	0
Dyspnea	5 (11)	0
Rash	5 (11)	0
Cough	5 (11)	0
Laboratory Abnormalities – no. (%)		
Alkaline phosphatase elevated	27 (59)	4 (9)
Alanine aminotransferase elevation	21 (46)	3 (7)
Anemia	21 (46)	3 (7)
Platelet count decreased	21 (46)	4 (9)
Aspartate aminotransferase elevation	18 (40)	3 (7)
Hypoalbuminemia	10 (22)	0
Hyperglycemia	10 (22)	5 (11)
Neutrophil count decreased	8 (17)	4 (9)
Hypokalemia	8 (17)	5 (11)
Hypomagnesemia	8 (17)	0
Hypocalcemia	8 (17)	0
White blood cell decrease	7 (15)	0

[1]Thirteen patients experienced an SAE: abdominal pain (2), Bile duct stenosis (1), small bowel obstruction (1), cellulitis (1), pneumonia (1), ileus (1), clostridium difficile colitis (1), nausea (1), vomiting (1), duodenal perforation (1), gastrointestinal bleed (2), cholangitis (1), cellulitis (1), peripheral vascular disease (1). Only nausea and vomiting in 1 patient were related to study treatment (grade 3).

© 2024 Cecchini M et al. *JAMA Oncology*.

eFigure 4. Mutational Signatures for Baseline ctDNA-Negative and ctDNA-Positive Cohorts

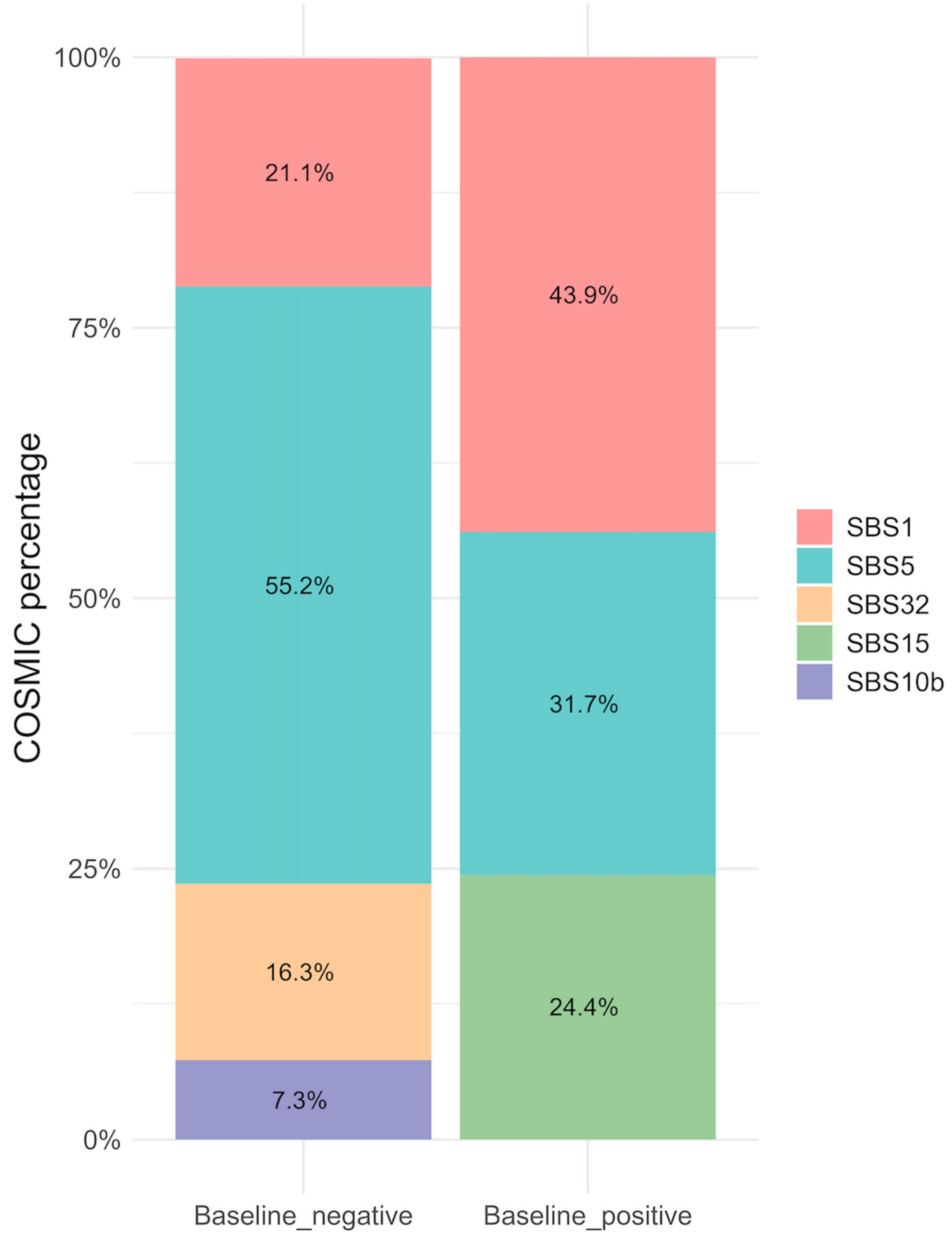

COSMIC mutational signatures for baseline ctDNA negative (left column) and baseline ctDNA positive (right column).

© 2024 Cecchini M et al. *JAMA Oncology*.

eFigure 5. Mutational Signatures for Exceptional, Intermediate, and Poor Responders

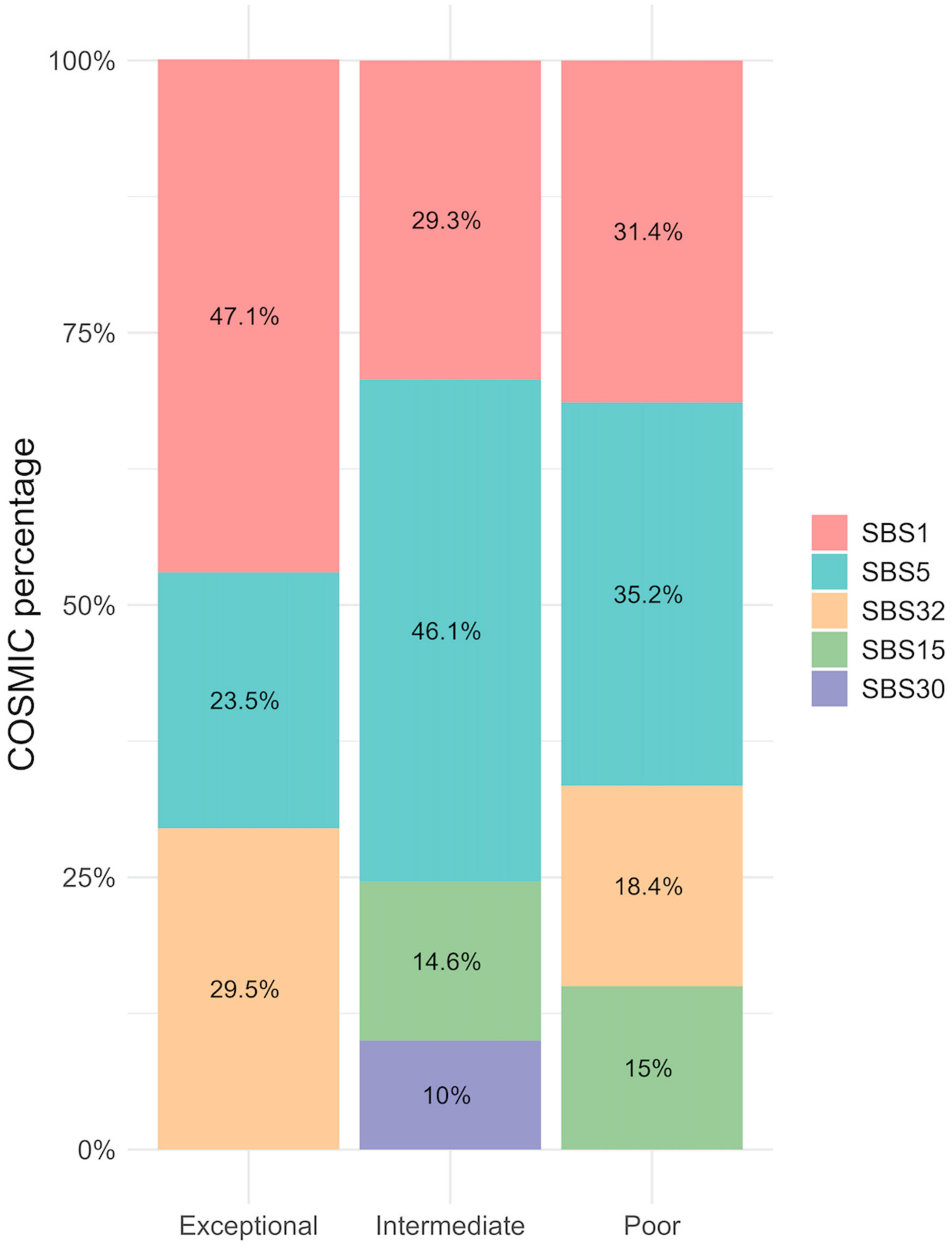

COSMIC mutational signatures for patients with exceptional survival (≥ 50 months), intermediate survival (25-49 months) and poor survival (<25 monhts).

© 2024 Cecchini M et al. *JAMA Oncology*.
