# Supplementary material for: Perioperative Modified FOLFIRINOX for Resectable Pancreatic Cancer: A Nonrandomized Controlled Trial
Source: JAMA Oncol. 2024 Jun 20;10(8):1027–35. doi: 10.1001/jamaoncol.2024.1575 (PMC11190830; doi:10.1001/jamaoncol.2024.1575)
Supplement: Supplement 3. — Data Sharing Statement [file jamaoncol-e241575-s003.pdf]

Data Sharing Statement

Cecchini. Perioperative Modified FOLFIRINOX for Resectable Pancreatic Cancer. *JAMA Oncol*. Published June 20, 2024. doi:10.1001/jamaoncol.2024.1575

Data
Data available: No

Additional Information
Explanation for why data not available: Patient consent did not include the public release of de-identified clinical data. The biomarker data may be made available to other investigators based on a reasonable request as discussed in our data sharing statement.
